# The Calmodulin Binding Region of the Synaptic Vesicle Protein Mover Is Required for Homomeric Interaction and Presynaptic Targeting

**DOI:** 10.3389/fnmol.2019.00249

**Published:** 2019-11-08

**Authors:** Asha Kiran Akula, Xin Zhang, Julio S. Viotti, Dennis Nestvogel, Jeong-Seop Rhee, Rene Ebrecht, Kerstin Reim, Fred Wouters, Thomas Liepold, Olaf Jahn, Ivan Bogeski, Thomas Dresbach

**Affiliations:** ^1^Institute for Anatomy and Embryology, University Medical Center Göttingen, Göttingen, Germany; ^2^Molecular Physiology, Institute of Cardiovascular Physiology, University Medical Center, Göttingen, Germany; ^3^Department of Molecular Neurobiology, Max Planck Institute of Experimental Medicine, Göttingen, Germany; ^4^Institute for Neuropathology, University Medical Center Göttingen, Göttingen, Germany; ^5^Proteomics Group, Max Planck Institute of Experimental Medicine, Göttingen, Germany

**Keywords:** synaptic vesicle, mover, TPRGL, calmodulin, presynaptic

## Abstract

Neurotransmitter release is mediated by an evolutionarily conserved machinery. The synaptic vesicle (SV) associated protein Mover/TPRGL/SVAP30 does not occur in all species and all synapses. Little is known about its molecular properties and how it may interact with the conserved components of the presynaptic machinery. Here, we show by deletion analysis that regions required for homomeric interaction of Mover are distributed across the entire molecule, including N-terminal, central and C-terminal regions. The same regions are also required for the accumulation of Mover in presynaptic terminals of cultured neurons. Mutating two phosphorylation sites in N-terminal regions did not affect these properties. In contrast, a point mutation in the predicted Calmodulin (CaM) binding sequence of Mover abolished both homomeric interaction and presynaptic targeting. We show that this sequence indeed binds Calmodulin, and that recombinant Mover increases Calmodulin signaling upon heterologous expression. Our data suggest that presynaptic accumulation of Mover requires homomeric interaction mediated by regions distributed across large areas of the protein, and corroborate the hypothesis that Mover functionally interacts with Calmodulin signaling.

## Introduction

At chemical synapses, neurotransmitter release occurs by exocytotic fusion of neurotransmitter storing organelles, called synaptic vesicles (SVs). These SVs are clustered at presynaptic sites of an axon. Upon exocytosis, they are locally recycled after retrieval from the presynaptic plasma membrane by endocytosis (Rizzoli, 2014). A network of presynaptic scaffolding proteins ascertains that SVs can only fuse at sites where this scaffold is assembled, i.e., at so-called active zones (Dresbach et al., 2001; Sudhof, 2012). In essence, three molecular events mediate transmitter release at presynaptic nerve terminals: scaffolding proteins tether SVs to the active zone, make them fusion-competent, and guarantee active zone integrity; SNARE proteins and SM (Sec1/Munc18-like) proteins mediate the fusion reaction; and synaptotagmin-1 acts as calcium sensor, making the fusion reaction calcium-dependent (Sudhof, 2013; Imig et al., 2014). The fusion reaction is highly conserved throughout evolution, including membrane fusion in yeast. Similarly, calcium dependence and scaffolding functions are highly conserved, as they are mediated by orthologous proteins in invertebrates, such as *C. elegans* and *Drosophila*, and vertebrates including mammals (Sudhof, 2012, 2013).

Intriguingly, some presynaptic proteins occur only in vertebrates, e.g., synuclein, or deviate unusually strongly from their invertebrate ancestors, e.g., the scaffolding proteins Bassoon and Piccolo, and the SV associated proteins DOC2 and Mover/TPRGL/SVAP-30. Bassoon and Piccolo are unusually large proteins, with molecular weights of 440 and 550 kDa, respectively. They are multi-domain proteins that bind numerous associated proteins, presumably to recruit regulators of exocytosis to the active zone (Cases-Langhoff et al., 1996; tom Dieck et al., 1998; Wang et al., 1999). In addition, they downregulate proteasomal and autophagial degradation of synaptic components, thus protecting synapses from disassembly, and contributing to their longevity (Waites et al., 2013; Okerlund et al., 2017). Likewise, Synucleins, in particular the alpha-Synuclein isoform, appear to protect SNARE proteins, and malfunction of Synucleins contributes to neurodegeneration in Parkinson’s disease (Burré et al., 2010; Burré, 2015). Doc2 is a calcium-binding protein displaying slower kinetics compared to synaptotagmin-1, consistent with its proposed role in regulating spontaneous and asynchronous transmitter release (Yao et al., 2011; Courtney et al., 2018). Mover is heterogeneously expressed among distinct types of synapses (Wallrafen and Dresbach, 2018). At the rat calyx of Held, it appears to modulate the calcium-sensitivity of release, thus affecting presynaptic release probability and short-term depression (Korber et al., 2015).

All five proteins lack a transmembrane region, suggesting that they are either soluble proteins or peripheral membrane proteins. In addition, functional domains and molecular properties are well-known for four of these proteins, e.g., coiled-coil domains in Bassoon and Piccolo, oligomerization features in Synucleins, and calcium-binding C2 domains in Doc2 (Friedrich et al., 2010; Gundelfinger et al., 2015; Lashuel et al., 2013), but less is known about the molecular properties of Mover. In a yeast-2-hybrid assay, we identified Mover, also called SVAP30 (Burré et al., 2006a,b) and TPRGL (Antonini et al., 2008) as a Bassoon binding protein (Kremer et al., 2007). Mover consists of 266 amino acids. Biochemical assays indicate that Mover is associated with membranes as a peripheral membrane protein, that phosphorylation affects its association with SVs, and that Mover binds to Calmodulin (CaM, Kremer et al., 2007; Ahmed et al., 2013; Korber et al., 2015). Primary structure analysis indicates that Mover contains a so-called hSac2 homology domain and a predicted Calmodulin-binding domain, but little is known about the significance of these domains and about the molecular properties of Mover in general.

To learn more about its properties, we had generated recombinant Mover constructs both for expression in yeast and in mammalian cells. So far, the most prominent features of recombinant full-length Mover are its tendency to self-interact in biochemical assays (Ahmed et al., 2013), and its accumulation in presynaptic terminals in cultured neurons (Kremer et al., 2007; Ahmed et al., 2013) and *in vivo* (Korber et al., 2015). Here, we have used existing and newly generated constructs to test the following questions: first, are there functional domains that mediate its best-characterized features, i.e., presynaptic targeting and self-interaction? Second, is phosphorylation at two distinct sites involved in self-interaction and/or presynaptic targeting? Third, what is the role of the few predicted functional domains, i.e., the hSac2 domain and the predicted Calmodulin-binding region?

## Materials and Methods

### Ethics Statement

All animal experiments were performed in accordance with the guidelines for the welfare of experimental animals issued by the State Government of Lower Saxony, Germany.

### Antibodies and Mammalian Expression Constructs

Antibodies: Mover (Synaptic Systems, 248003); Tubulin (Sigma, DM1A); myc-tag (Santa Cruz, clone 9E10); Synapsin-1 (Synaptic Systems, clone 46.1); Synaptophysin (Sigma, SVP-38); GFP (Abcam, cat. GFP6556, for immunofluorescence); GFP (Synaptic Systems, 132002, for Western blots); Bassoon (ENZO Life Sciences, sap7f407). Secondary antibodies (Invitrogen): anti-mouse Alexa 647 1:1,000; anti-mouse Cy3 1:500; anti-guinea pig Alexa 647 1:1,000; anti-rabbit Alexa 647 1:1,000. All mammalian expression constructs carried the CMV promotor. All recombinant Mover sequences were from rat Mover. For tagging with GFP, the A206K mutant of EGFP was used, which prevents dimerization of EGFP.

### Co-immunoprecipitation

Co-immunoprecipitation was performed as described (Ahmed et al., 2013). Briefly, transfected HEK293 cells were harvested in IP-Lysis buffer (50 mM Tris-HCl pH 7.5; 150 mM NaCl; 2 mM EDTA; 0.5% NP40; Complete protease inhibitor (Roche). After centrifugation at 15,000 *g* for 10 min lysates were preincubated with 10 ml of Protein A/G sepharose beads for 1 h at 4°C. The lysates were then incubated with monoclonal anti-myc antibodies for 1 h at 4°C. Protein A/G was added, and the mixture was incubated on a shaker over night at 4°C. The beads were pelleted at 5,000 g for 30 s and washed three times with IP-lysis buffer. Bound proteins were eluted by incubation for 10 min at 95°C in SDS sample buffer. The samples were analyzed by SDS-PAGE and Western blotting.

### Preparing and Transfecting Primary Cultures of Rat Hippocampal Neurons for Targeting Experiments

Hippocampi were obtained from E19 rats or postnatal day 0 (P0) mice. They were treated with trypsin for 20 min at 37°C, triturated to dissociate the cells, plated at 25,000–50,000 cells/cm^2^ on poly-lysine coated coverslips, and cultured in Neurobasal supplemented with 2% B-27 and 2 mM Glutamax (Gibco/Invitrogen). Neurons growing on 12 mm coverslips in 24-well plates were transfected with calcium phosphate at 3–4 DIV as described previously (Dresbach et al., 2001; Ahmed et al., 2013). Before transfection, medium was removed, saved, and replaced with 500 μl 37°C Optimem (Life Technologies) and incubated for 30–60 min. 105 μl transfection buffer (274 mM NaCl, 10 mM KCl, 1.4 mM Na_2_HPO_4_, 15 mM glucose, 42 mM HEPES, pH 7.06) was added dropwise to a solution containing 7 μg of DNA and 250 mM CaCl_2_, with gentle vortexing. This mixture was incubated for 20 min at room temp, 30 μl was added per well, and neurons incubated for 60–90 min. This medium was then removed, cells were washed 3× in 37°C Neurobasal medium, and saved medium added back to the transfected cells. Cultures were fixed on DIV 14 using 4% paraformaldehyde and immunostained.

### Preparing Primary Cultures of Mouse Hippocampal Neurons for Electrophysiology

For electrophysiological analysis of mass cultures, dissociated cultured hippocampal neurons were prepared from wildtype (WT) and KO mice of P0. The cultures were prepared in a similar fashion to the method described by Burgalossi et al. (2012), but with several modifications for dissociated instead of autaptic cultures. Hippocampi were dissected from newborn mice in cold Hank’s Balanced Salt Solution (Gibco, Thermo Fischer Scientific) and digested by papain (25 U/ml) for 60 min at 37°C under gentle agitation (450 rpm). Digestion was then stopped by substitution of the papain solution by a trypsin inhibitor-based stop solution and hippocampi were incubated in this solution for 15 min at 37°C with gentle agitation (450 rpm). Washing out of stop solution was followed by trituration of the tissue using a 200 μl pipette tip. Neurons were then counted and plated at a density of 25,000 cells per well in a 24-well dish filled with Neurobasal A medium supplemented with B27, GlutaMax and penicillin/streptomycin (0.5 ml/L; all from Gibco, Thermo Fischer Scientific).

For electrophysiological analysis of microisland cultures, hippocampal neurons were prepared and cultured as described previously (Burgalossi et al., 2012). In brief, astrocytes for microisland cultures were obtained from mouse cortices from P0 WT animals using digestion with 0.25% trypsin (Gibco) for 20 min at 37°C. The cells were plated in T75 culture flasks in DMEM medium (Gibco) containing 10% FBS (PAA) and Penicillin/Streptomycin (Gibco). The medium was exchanged the day after plating, and cells were allowed to grow for 7–10 days. Following this, cells were collected from the flask using trypsin digestion and plated at a density of 12,000 cells/well on 32 mm coverslips. The coverslips used for microisland cultures were first coated with agarose (Sigma-Aldrich), and then with a coating solution containing poly-D-lysine (Sigma-Aldrich), acetic acid, and collagen (BD), using a custom-made stamp to generate 200 μm × 200 μm substrate islands. Hippocampi from P1 mouse were dissected free of meninges and separately collected in ice-cold Hanks Buffered Salt Solution (HBSS; Gibco). They were incubated in papain solution containing 2 mg cysteine, 10 ml DMEM (Gibco), 1 mM CaCl_2_, and 0.5 mM EDTA, along with 20–25 units of papain (Worthington Biomedical Corporation); 45 min for hippocampi and 60 min for striatum at 37°C). After washing, cells were triturated and counted in a Fuchs-Rosenthal or Naubauer chamber. The cells were plated in pre-warmed Neurobasal medium (Gibco) supplemented with B-27 (Gibco), glutamax (Gibco) and Penicillin/Streptomycin (Gibco) at a density of 25,000 cells/well on 18 mm coverslips for high density cultures or 4,000 cells/well on a 32 mm coverslip for microisland cultures.

### Microscopy and Image Analysis

Neurons were fixed with 4% PFA in PBS for 20 min and permeabilized using PBS with 0.3% Triton X-100, 2% BSA, 10% FCS, and 5% glucose. All antibodies were diluted in permeabilization buffer. Microscope images were acquired using a CoolSNAP HQ2 CCD camera (Photometrics) attached to a Zeiss AxioObserver Z1 with a Plan ApoChromat NA 1.4 40× oil objective. Exposure time for the images was kept consistent for each experiment. Synaptic puncta were detected using MetaMorph software. They were selected automatically after setting a threshold. The threshold was set to 400, and then kept constant for all images. Statistical analysis was performed using Prism (GraphPad Software).

### Statistical Analysis

For quantitative analysis of microscopy images, we used the following definition for “independent experiment”: to obtain rat cultured neurons for one experiment, one pregnant rat was sacrificed, and the hippocampi from the embryos obtained from this rat were pooled starting with the trypsin treatment. The coverslips obtained from plating the resulting neurons constitute one experiment. To obtain mouse cultured neurons, P0 pups were sacrificed, and the two hippocampi of each pup were processed while genotyping was initiated. Culturing and analyzing the neurons of one WT pup and one littermate knockout pup constitutes one experiment. We analyzed neurons obtained from pups of at least three litters. After data acquisition, we first employed the D’Agostino–Pearson omnibus normality test to test if the data followed a Gaussian distribution and afterward a two-tailed student’s *t*-test or a one-way ANOVA test were performed to test for significant differences, as indicated in the results or legends. Error bars indicate standard error of the mean.

### KO Generation and Genotyping

All mice (*Mus musculus*) were bred and maintained at the central animal facility of the University Medical Center, Göttingen. Embryonic stem cells (129/Ola) harboring the recombined Mover locus were generated by PolyGene (Switzerland), and injected into blastocysts. The Neo cassette was removed by crossing with a Flp deleter line (Farley et al., 2000). The resulting floxed mice were crossed with the E2A-Cre mouse line (Lakso et al., 1996) to generate a global knockout. Mice were genotyped by PCR using the following primers to amplify genomic DNA sequences: forward primer P1 (5′-ccaatcacaaggcgaacgag-3′); forward primer P2 (5′-cattcagtgggacaagcaga-3′); reverse primer P3 (5′-cttggatcaggagagccttg-3′). The PCR reaction was carried out for 40 cycles with denaturation at 95°C for 30 s, annealing at 56°C for 1 min, and extension at 72°C for 1 min. WT and KO animals were identified by the presence of a specific 867 bp and a 697 bp band, respectively (see Supplementary Figure S5). The floxed Mover mouse line will be available from The Jackson Laboratory as JAX#032466.

### Brain Homogenates

For each homogenate one mouse cortex was transferred to a glass Teflon homogenizer and homogenized using 1.5 ml of homogenization buffer (0.32 M sucrose, 1 mM NaHCO_3_) with freshly added protease inhibitor cocktail and Benzonase (Sigma). Homogenization was performed using 10 strokes at 1,200 rpm on ice. Cell debris and nuclei were pelleted for 10 min at 1,000 g, and the supernatant was processed further. After determining the protein concentration by the BCA assay, the supernatant was analyzed by Western blotting.

### FRET Analysis of the Interaction Between GFP-Mover and RFP-Mover

FRET was measured by Fluorescence Lifetime Imaging (FLIM) using time-correlated single photon counting on an Olympus FV1000 confocal microscope, equipped with FLIM hardware from Picoquant (Picoquant, Berlin): picosecond-pulsed laser diodes, single-photon avalanche diode detectors (Micro Photon Devices, PDM series), and PicoHarp 300 counting electronics.

FLIM recordings of the GFP signal were acquired with a 63× NA 1.35 UPLS-Apo objective at am image size of 256 × 256 pixels and not exceeding count rates of 300 kcounts/s. GFP was excited using a LDH-D-C-458 picosecond pulsed laser diode at a repetition rate of 40 MHz. Lifetime fitting was performed from the individual photon arrival times by the TRI2 program using the mono-exponential phasor method (Barber et al., 2009). Lifetime distributions of five images were normalized, averaged, and transformed to FRET efficiencies using *FRET = (τ_D_ − τ_DA_)/τ_D_*, in which τ_D_ represents the average lifetime in the absence of FRET, i.e., as measured in an experiment in which the FRET donor GFP-Mover was expressed without co-expression of the acceptor RFP-Mover, and τ_DA_ represents the average lifetime in the presence of FRET, i.e., in cells co-expressing GFP- and RFP-Mover. The distributions were fitted with a Gaussian function (Igor Pro, Wavemetrics, Portland, Lake Oswego, OR, USA). FRET efficiency images are displayed as GFP intensity-weighed lifetime images in a false-color lookup table (Hinman and Sammak, 1998) using FIJI (Schindelin et al., 2012). Lifetime distributions were acquired in five different fields of view (*n* = 5, from two experiments) of axonal regions containing synaptic puncta. The complete data set contains hundreds of synaptic puncta with co-localizing GFP and RFP signals per experimental condition.

### Photoaffinity Labeling (PAL)-Based Competition Assay

Photoreactive peptides were synthesized by using standard solid-phase fluorenylmethoxycarbonyl (Fmoc) chemistry and the amino acid derivative Fmoc-para-benzoylphenylalanine (Bpa, Novabiochem; Jahn et al., 2002). PAL-based competition experiments with recombinant CaM were performed and analyzed as described previously (Dimova et al., 2006; Lipstein et al., 2012). Briefly, 5 μM CaM and 5 μM Bpa-Mover(203-221) were incubated in 10 mM HEPES (pH 7.2), 150 mM KCl, 5 mM DTT for 2 h at RT in the dark with (100 μM Ca^2+^) or without (2 mM EGTA) calcium, and in the presence of increasing concentrations (0–250 μM) of Mover(203-221) or its mutant variants. Following UV irradiation, photoadduct formation was visualized and quantified by SDS-PAGE and linear matrix-assisted laser desorption/ionization time-of-flight mass spectrometry (MALDI-TOF-MS).

### NFAT1 Translocation Assay

Imaging experiments were performed with a Zeiss Cell Observer Z1 equipped with a 40× oil Fluar (N.A. 1.3) objective, multi-filter system, fast acquisition EMCCD camera (Evolve^®^512 Delta) and LED system (Colibri, Zeiss) at 37°C. Data were analyzed with AxioVision software (Zeiss, Oberkochen, Germany). HeLa cells were cultivated in DMEM medium (Gibco^TM^, Cat: 11965084) supplemented with 10% FCS at 37°C in 5% CO_2_. About 2.5*10^5^ HeLa cells were seeded on 25 mm round glass coverslips for 12 h before transfection of DNA constructs. Mover-IRES-mKate, Mover F206R-IRES-mKate or Mover4mut-IRES-mKate and NFAT1-GFP plasmid were co-transfected using Fugene^®^ HD (Promega GmbH, Mannheim, Germany) and Opti-MEM^TM^ (Gibco^TM^, Cat: 31985070) along with 1 μg of plasmid DNA according to the manufacturer’s instructions, 48 h before the imaging experiment. During the experiment, HeLa cells were perfused with Ringer’s buffer containing 1 mM Ca^2+^ and activated by 10 μM histamine (Sigma, H7125), as indicated. NFAT1-GFP fluorescence was recorded using LED diode (excitation 505 nm) and emission filter (525 ± 25 nm). The increase of fluorescence intensities in the nucleus was marked with region of interest (ROI) and analyzed by normalizing the background-corrected fluorescence intensity. Mann–Whitney test was used for testing the significance of the results.

### Electrophysiological Analysis of Dissociated Mass Cultures

Whole-cell patch clamp recordings were made at 37°C ± 0.5°C using a HEKA EPC-10 USB amplifier and the software Patchmaster (HEKA Electronics). During recordings, cultures were continuously perfused with extracellular solution consisting of (in mM): NaCl 125.0, KCl 2.5, NaHCO_3_ 25.0, NaH_2_PO_4_ 1.25, CaCl_2_ 2.0, MgCl_2_ 1.0, glucose 20.0 (pH 7.4); with the addition of 1 μM tetrodotoxin (TTX). The intracellular solution consisted of (in mM): 150 K^+^-D-Gluconate, 10 NaCl, 3 Mg-ATP, 0.3 Na-GTP, 10 Hepes, and 0.05 EGTA (pH 7.3). Cells were voltage-clamped at −70 mV and a liquid junction potential of 13 mV was corrected online. Recordings were discarded if initial series resistance was greater than 10 MΩ. Recordings were digitized at 20 kHz and filtered at 2.9 kHz. Miniature post-synaptic currents were detected by the software MiniAnalysis (Synaptosoft).

### Electrophysiological Analysis of Autaptic Cultures

Whole-cell patch clamp recordings were performed in autaptic hippocampal neurons as described before (Burgalossi et al., 2012; Nair et al., 2013). Neurons were voltage clamped at −70 mV using a Multiclamp 700 B amplifier and pClamp software (Molecular Devices). Only cells with a series resistance of ≤ 12 MΩ were included in the analysis. The series resistance was compensated by 20–70% and the data was acquired at a sampling rate of 10–25 kHz. Neurons were maintained in fresh extracellular bathing solution throughout the recordings and a fast perfusion system (SF-77, Warner Instrument) was used to apply pharmacological agents. The RRP size was measured by applying 500 mM sucrose solution for 6s as published previously (Nair et al., 2013). Spontaneous mEPSCs were measured in the presence of 300 nM and AP-evoked EPSCs were measured by clamping the membrane potential for 2 ms from −70 mV to 0 mV. The following solutions were used for experiments. Intracellular solution: 136 mM KCl, 17.8 mM HEPES, 15 mM Phosphocreatine, 1 mM EGTA, 0.6 mM MgCl2, 0.3 mM Na-GTP, 4 mM Mg-ATP, 5 U/mL Creatinephosphokinase (solution adjusted to pH 7.4; ~320 mOsmol/L). Extracellular solution: 140 mM NaCl, 4 mM CaCl2, .4 mM KCl, 10 mM HEPES, 24 mM MgCl2, 10 mM Glucose (solution adjusted to pH 7.3, ~310 msOsmol/L).

Whole-cell recordings in cultured autaptic hippocampal neurons were carried out as described before (Burgalossi et al., 2012; Nair et al., 2013). In brief, cells were voltage clamped at −70 mV using a Multiclamp 700 B amplifier under the control of the pClamp software (Molecular Devices). The data were sampled at a rate of 10–25 kHz and the series resistance was compensated by 20%–70%. Only cells with series resistances ≤12 MΩ were included in the analysis. In order to measure AP-triggered EPSCs, the membrane potential was depolarized for 2 ms from −70 mV to 0 mV. Cultured neurons were constantly supplied with fresh extracellular bathing solution *via* a perfusion system. Pharmacological agents were applied using a fast flow application system consisting of valve-controlled capillaries and a stepper device (SF-77, Warner Instrument). Spontaneously occurring mEPSCs were measured in the presence of 300 nM TTX and the RRP size of neurons was determined by applying 500 mM sucrose solution for 6–8 s as published previously (Rosenmund and Stevens, 1996; Basu et al., 2007; Jockusch et al., 2007). The following solutions were used for experiments. Intracellular solution: 136 mM KCl, 17.8 mM HEPES, 15 mM Phosphocreatine, 1 mM EGTA, 0.6 mM MgCl_2_, 0.3 mM Na-GTP, 4 mM Mg-ATP, 5 U/mL Creatinephosphokinase (solution adjusted to pH 7.4; ~320 mOsmol/L). Extracellular solution (“Base+”): 140 mM NaCl, 4 mM CaCl_2_, 0.4 mM KCl, 10 mM HEPES, 24 mM MgCl_2_, 10 mM Glucose (solution adjusted to pH 7.3, ~310 msOsmol/L).

## Results

**Figure 1:**
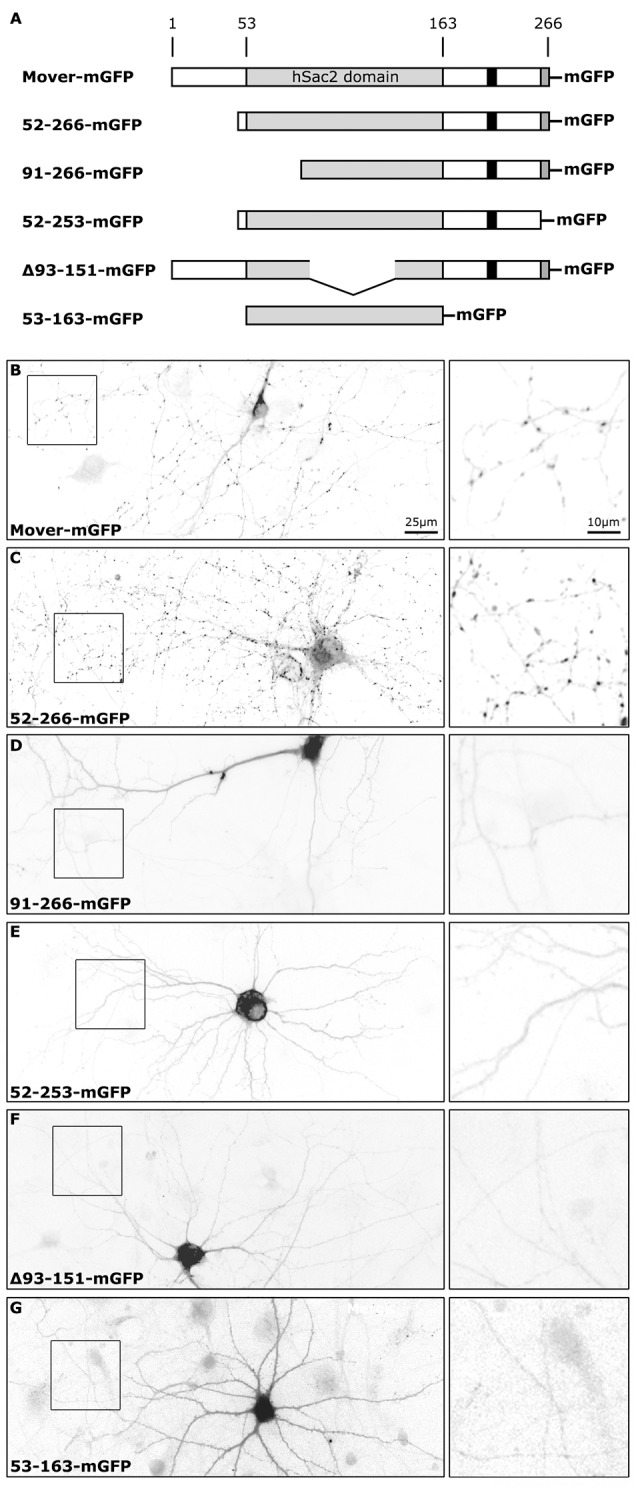
Subcellular distribution of recombinant Mover variants in cultured hippocampal neurons. **(A)** Schematic depicting the proteins tested. The numbers indicate amino acids in the primary structure of rat Mover. The black bar indicates the location of the Calmodulin (CaM) binding region. All proteins are tagged with monomeric EGFP (mGFP). **(B–G)** Inverted gray level epifluorescence images representing the localization of GFP-tagged rat Mover constructs in DIV14 rat hippocampal neurons. The square panels on the right represent zooms of the boxes. Mover-mGFP and 52-266-mGFP are known to accumulate at synapses and produce the corresponding punctate staining pattern. All other variants are homogeneously distributed throughout the cytoplasm, indicating that they fail to accumulate at synapses. A 20× objective was used for the overviews on the left, in order to capture an entire neuron. The exposure time was set to visualize fluorescence in the neurites. Using these setting, nuclear and somatic fluorescence is saturated, because these compartments have much bigger volume than neurites. The distribution of each construct was determined in more than 30 neurons on a total of six coverslips from three independent experiments.

To investigate the targeting properties of recombinant Mover we compared three newly generated constructs, called 52-253-mGFP, Δ93-151-mGFP and 53-163-mGFP, to three previously studied constructs (Figure 1A). All constructs carry C-terminal mGFP tags, representing a monomeric variant of EGFP. We had previously observed that Mover constructs either comprising all 266 amino acids (termed Mover-mGFP) or lacking the amino terminal 51 amino acids (termed 52-266-mGFP) were targeted to presynaptic terminals in transfected cultured neurons. In contrast, removing the amino terminal 90 amino acids abolished presynaptic targeting in a construct called 91-266-mGFP, indicating that the sequence spanning amino acids 51 through 90 is required for presynaptic targeting (Ahmed et al., 2013). As expected, expressing Mover-mGFP and 52-266-mGFP produced a punctate fluorescence pattern whereas 91-266-mGFP was diffusely distributed and failed to produce any puncta, indicating that the construct does not accumulate at synapses (Figures 1B–D). To quantify the accumulation of Mover-mGFP and 52-266-mGFP at synapses we immunostained transfected cultures with antibodies against the presynaptic marker Synaptophysin and the dendrite marker MAP2 (Supplementary Figure S1). Our criteria for synaptic localization was that a GFP-punctum had to colocalize with Synaptophysin and—to make the selection even more stringent—had to be located near a dendrite. We then determined the average GFP-fluorescence intensity of 60 such puncta and divided it by the average GFP-fluorescence intensity observed in 60 non-synaptic axon regions. The resulting ratio was 10.94 for Mover-mGFP, 8.84 for 52-266-mGFP, and 9.37 for GFP-VAMP, another major component for SVs (Supplementary Figure S1), indicating that the two Mover constructs capable of presynaptic targeting were enriched at synapses to the same extent as GFP-VAMP. All other constructs also failed to produce any punctate signals (Figures 1E–G). The absence of any punctate GFP-signals in neurons expressing these constructs was obvious from low magnification images. To further corroborate this observation, we immunostained the cultures for Synaptophysin and MAP2 and identified axons as MAP2-negative processes (Supplementary Figure S2). Again, punctate accumulations were never observed in any axon. To provide a quantitative estimate of this lack of accumulation we determined the ratio of GFP-fluorescence at 15 axonal sites contacting dendrites to the GFP-fluorescence at 15 random sites that did not contact any dendrite. The resulting ratios never exceeded 2.61, and the average ratio was 1.6. This provides several insights: first, not only amino acids 51 through 90, but also the 13 most C-terminal amino acids (amino acids 254–266) are required for presynaptic targeting; second, a central portion of the hSac2 domain, which is lacking in Δ93-151-mGFP, is also required for presynaptic targeting; third, the hSac2 domain, which is represented by 53-163-mGFP, is not sufficient for presynaptic targeting. Taken together, these results indicate that several regions distributed all over the primary structure of Mover, are each required for presynaptic targeting.

**Figure 2:**
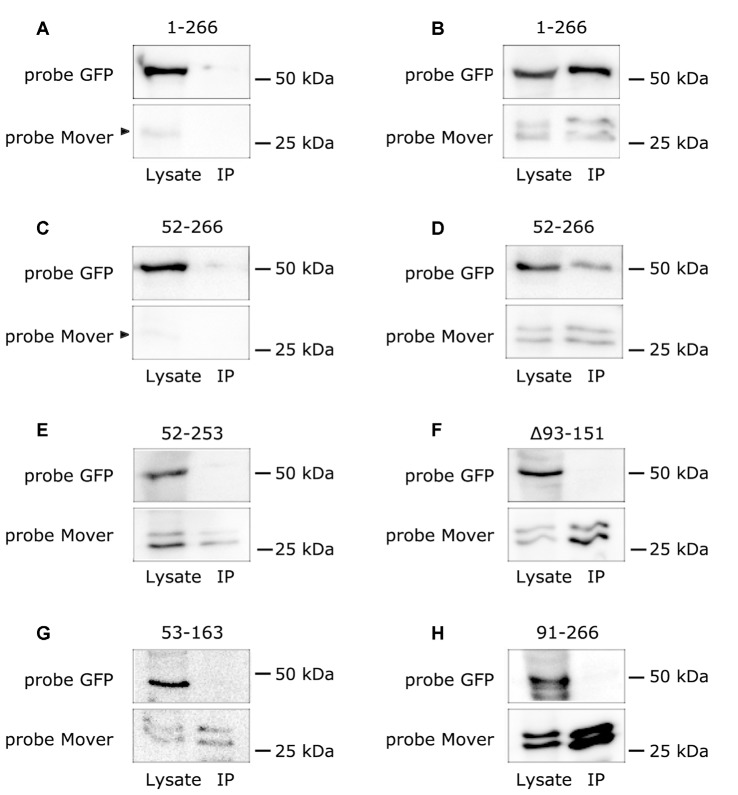
Mover variants that fail to target fail to undergo homomeric interaction with full-length Mover. The figure shows Western blots analyzing the results of immunoprecipitation of Mover variants expressed in HEK293 cells. **(A–H)** Each GFP-tagged Mover variant (indicated on top of each panel) was separately co-expressed with myc-tagged Mover. Sepharose-coupled antibodies against the myc epitope were used to pull down protein complexes. The 30 kDa region of the blot was probed with anti-Mover, to verify immunoprecipitation of Mover-myc, which runs as a double band. Panels **(A,C)** are controls where the GFP-tagged constructs were expressed without Mover-myc, showing that the GFP tagged constructs expressing amino acids 1-266 and 52-266 display only residual binding to Sepharose beads. The arrowheads indicate degradation products of the GFP-Mover constructs detected by the Mover antibody. “Lysate” indicates samples obtained before adding anti-myc antibodies, “IP” indicates samples of the immunoprecipitation pellet. The regions of the blot corresponding to the molecular weights of the GFP-tagged proteins were probed with anti-GFP. Only full-length Mover-mGFP (“1-266”) and the variant lacking the amino terminal 51 amino acids (“52-266”) co-immunoprecipitate with Mover-myc. All others fail to co-immunoprecipitate. The data represent two independent experiments, i.e., two HEK293 cell transfections followed by lysis and co-immunoprecipitation.

A prominent feature of full-length Mover is its tendency to self-interact (Ahmed et al., 2013). To test whether presynaptic targeting may be mediated by its self-interaction property, we aimed to determine which of the constructs co-immunoprecipitates with full-length Mover. To this end, we first characterized recombinant Mover expressed in HEK293 cells. Western blotting of HEK293 cell lysates revealed that the constructs displayed the expected apparent molecular weights, both when probed with a GFP antibody and when probed with the Mover antibody (Supplementary Figure S3). In addition to bands running in the expected range, GFP-fusion proteins expressing amino acids 1–266 or 52–266 of Mover produced immunosignals in the 30 kDa range, probably representing proteolytic cleavage of a fraction of the expressed proteins. Moreover, full-length Mover with a c-terminal myc-tag ran as a double band, perhaps representing posttranslational modification of this construct in HEK293 cells. Both bands ran at higher molecular weights than untagged Mover (Supplementary Figure S3) and were immunoprecipitated by an anti-myc antibody (Figure 2), suggesting that the smaller band is not a proteolytic degradation product and that this construct can be used for co-immunoprecipitation experiments. The Mover antibody also revealed a ca. 30 kDa band in untransfected HEK293 cells, perhaps representing FAM79A, the human Mover ortholog.

We then co-expressed full-length Mover-myc with either of six GFP-constructs in HEK293 cells. We used an anti-myc antibody to immunoprecipitate Mover-myc, and we used an anti-GFP antibody to test whether or not the GFP-constructs were co-immunoprecipitated. In addition, we verified that Mover-myc was present in the lysate and the immunoprecipitation pellet using an anti-Mover antibody. Interestingly, the two constructs that are capable of presynaptic targeting were also co-immunoprecipated with Mover-myc, whereas the four constructs that did not target were not co-immunoprecipitated (Figure 2). This indicates that disrupting Mover either at an N-terminal, C-terminal or central region abolishes both targeting and self-interaction. Only amino acids 1–51 can be removed without affecting these features.

**Figure 3:**
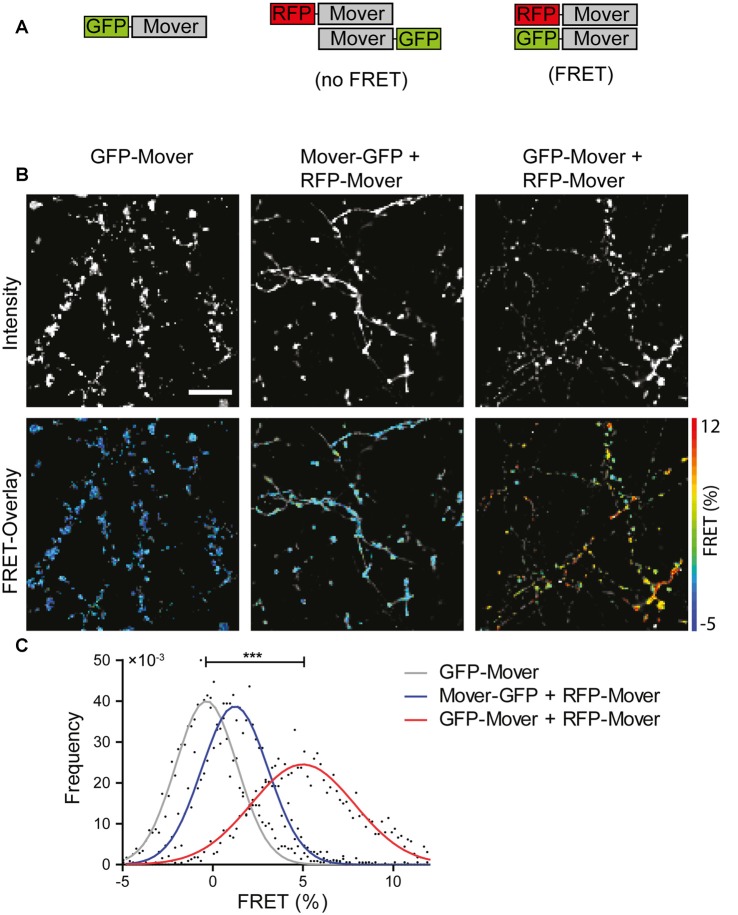
Homomeric interaction of recombinant Mover in cultured hippocampal neurons revealed by FRET analysis. **(A)** Schematic representation of the constructs and the result of FRET analysis. A construct encoding amino acids 52-266 of Mover was tagged either at its N-terminus or at its C-terminus with the fluorescent proteins indicated. **(B)** Representative intensity images (top row, gray scale) and FRET images overlayed with the corresponding intensity images (bottom row, false color). **(C)** FRET distribution per condition. *n* = 5 regions of interest (ROIs), *N* = 2 independent experiments, two-sided student’s *t*-test, ***p ≤ 0.001. Scale bar is 10 μm.

Self-interaction of Mover prominently occurs when Mover is expressed in non-neuronal cells. In order to investigate whether Mover also self-interacts when expressed in neurons, and to gain insight into the topological organization of the interacting partners, we performed FRET imaging on a mGFP-donor- and mRFP-acceptor-labeled FRET pair of Mover proteins. To this end, we generated two novel constructs comprising amino acids 52–266, i.e., the shortest version of Mover that still targets to synapses (Ahmed et al., 2013; and Figure 1). These new constructs either contained mGFP or mRFP immediately upstream of amino acid 52, and are called mGFP-52-266 and mRFP-52-266, respectively. It is not possible to use N-terminally tagged full-length Mover because these constructs aggregate upon expression (Ahmed et al., 2013). We transfected cultured hippocampal neurons either with mGFP-52-266 alone, with mRFP-52-266 and 52-266-mGFP (i.e., two constructs tagged at opposite ends), or with mRFP-52-266 and mGFP-52-266 (i.e., two constructs tagged at the same end). FRET microscopy of the punctate fluorescence signals produced by these proteins was performed by FLIM of the GFP donor signal (Figure 3). A statistically significant FRET signal was obtained only in puncta containing both mRFP-52-266 and mGFP-52-266 fluorescence. This shows that these two constructs physically interact as the fluorescent proteins are close enough to exhibit FRET, corroborating the notion that Mover indeed undergoes homomeric interaction at synapses. Moreover, as Mover proteins tagged at opposite ends with mGFP and mRFP do not show FRET, the N- and the C-termini are farther away from each other within the homomeric complex than the N-termini.

**Figure 4:**
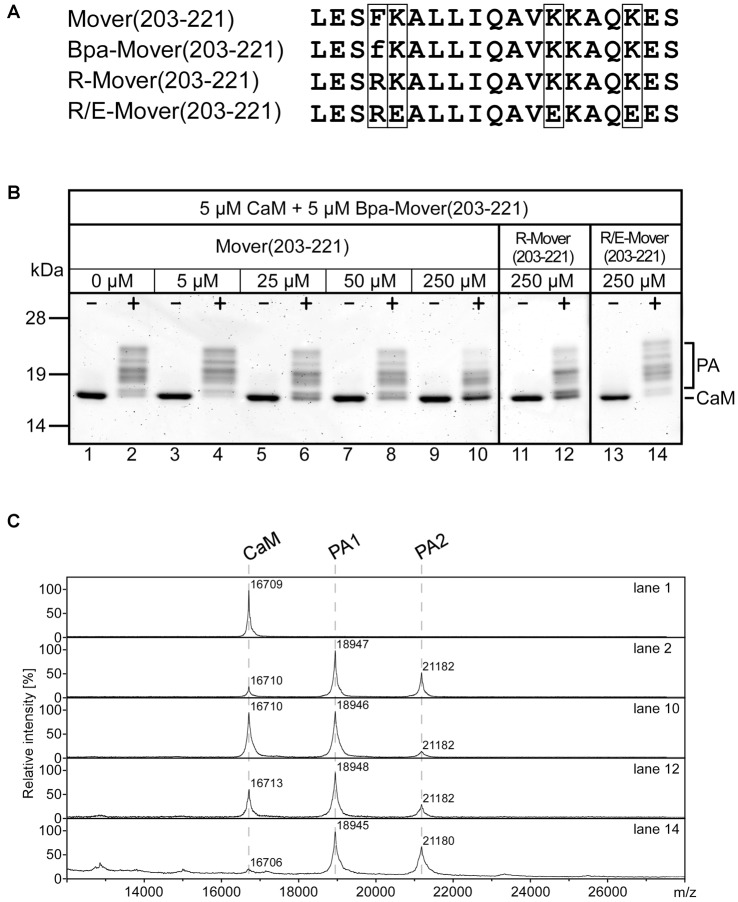
CaM-binding properties of wildtype (WT) and mutated Mover peptides. **(A)** Amino acid sequences of Mover(203-221), the photoreactive variant Bpa-Mover(203-221), the point mutant R-Mover(203-221) carrying an Arg residue instead of the hydrophobic anchor residue Phe, and the CaM-binding deficient R/E-Mover(203-221) carrying multiple mutations of basic residues within the CaM-binding sequence in addition. The sequence positions for amino acid exchanges are boxed. f, p-benzoylphenylalanine (Bpa). For photoaffinity labeling (PAL)-based competition assays, 5 μM CaM was incubated with 5 μM Bpa-Mover(203-221). Photoreactions were analyzed by gel electrophoresis **(B)** and MALDI-TOF-MS **(C)** to monitor the formation of covalent photoadducts (PA) in the mass range of 19 kDa (PA1, 1:1 peptide/CaM complex) to 21 kDa (PA2, 2:1 peptide/CaM complex). Photoreactions were performed in the absence (−) and presence (+) of 100 μM Ca^2+^, and with increasing concentrations of Mover(203-221), R-Mover(203-221), and R/E-Mover(203-221) as competitors. For the mutant peptides, only the highest concentration [50-fold molar excess over Bpa-Mover(203-221)] is shown. As can be followed most clearly by means of the intensity of the signal for free CaM, Mover(203-221) effectively suppressed photoadduct formation, while the mutant competitors R-Mover(203-221) and R/E-Mover(203-221) showed reduced or no affinity to CaM, respectively. Note that also a 50-fold molar excess of Mover(203-221) did not lead to full suppression of photoadduct formation. This was most likely due to the known positive correlation between bulkiness/hydrophobicity of N-terminal anchor positions in amphipathic CaM-binding peptides and their affinity for CaM (O’Neill et al., 1989; Dimova et al., 2006). Accordingly, Bpa-Mover(203-221) with a Bpa anchor residue (two phenyl moieties) binds CaM with a higher affinity than Mover(203-221) with a Phe anchor residue (one phenyl moiety). Two independent experiments were performed.

Another feature of Mover, in addition to its accumulation at presynaptic sites and its self-interaction, is its binding to CaM. Both recombinant and endogenous Mover bind to recombinant CaM in a pull-down assay (Korber et al., 2015). Primary structure analysis with computational prediction tools (Yap et al., 2000) revealed that amino acids 203-221 of rat Mover contain candidate sequences for CaM-binding (Figure 4A). Apart from a less well-defined Ca^2+^-independent IQ-like motif (amino acids 211/212), this sequence stretch is characterized by hydrophobic amino acids in a particular spacing (1-5-8 when F206 is considered as primary hydrophobic anchor residue) and several positively charged residues (K207, K215, K216 and K219), indicative of a Ca^2+^-dependent CaM-binding motif (Lipstein et al., 2017). As this motif resembles the functional CaM-binding motifs established in the Munc13 family of presynaptic scaffolding proteins (Junge et al., 2004; Lipstein et al., 2012, 2013), we decided to test whether this region of Mover is able to bind to CaM by using a PAL-based competition assay. In this assay, photoreactive CaM-binding peptides and CaM upon UV irradiation form a covalent heteromeric photoadduct (~19 kDa), which can be differentiated from monomeric CaM (~17 kDa) by its molecular weight using gel electrophoresis or mass spectrometry (MS; Dimova et al., 2006; Lipstein et al., 2012). For this purpose, we generated the peptide Mover(203-221) and its photoreactive variant Bpa-Mover(203-221), in which p-benzoylphenylalanine (Bpa) replaces the phenylalanine at position 206 to enable photo-cross-linking reactions with CaM (Figure 4A). While no photoadducts were formed when equimolar amounts of Bpa-Mover(203-221) and CaM were incubated in the absence of Ca^2+^ (lane 1 in Figure 4B), nearly all of the CaM was converted into photoadduct species in the presence of Ca^2+^ (lane 2 in Figure 4B), indicating that the interaction is strictly Ca^2+^-dependent. Heterogeneous appearance of photoadduct bands in the gel is occasionally seen in this assay (Junge et al., 2004; Dimova et al., 2006; Lipstein et al., 2012), and may be explained by changes in electrophoretic mobility of the adducts when photocross-linking occurred at slightly different sites. In line with this notion, MS revealed the presence of a 1:1 peptide-CaM-photoadduct as the dominant form, but a 2:1 stoichiometry was also apparent (~21 kDa), at least to some extent (mass spectra corresponding to lane 1 and 2 in Figure 4C). However, based on our experience with photoreactive CaM-binding peptides derived from other protein families including Munc13 (see Lipstein et al., 2017 and references therein), we consider these 2:1 complexes as artifacts likely resulting from the use of short (~20 amino acid) minimal CaM-binding sequences in combination with high Ca^2+^ concentrations and long irradiation times.

To verify the specificity of photoadduct formation, increasing amounts of Mover(203-221) were included as competitor into the photoreaction. Because of the heterogeneity of the photoadduct species with regard to electrophoretic mobility and stoichiometry, the decline of photoadduct signal was followed by means of the re-appearing signal for free CaM and was found to correlate with the amount of competitor added (lanes 4, 6, 8, 10 in Figure 4B and mass spectrum corresponding to lane 10 in Figure 4C). The ability of Mover(203-221) to suppress photoadduct formation indicated that photoreactive and unmodified form of the peptide bind to the same binding site on CaM.

We next designed mutant peptides to identify the amino acid residues critical for CaM binding with the ultimate aim of generating a Mover variant that does not bind to CaM. It was shown earlier for Munc13-1, that replacing the hydrophobic anchor residue in position 1 of the CaM-binding motif by an Arg residue completely abolishes the interaction with CaM (Junge et al., 2004; Lipstein et al., 2013). However, in our competition assay, the Mover peptide carrying the corresponding point mutation F206R [referred to as R-Mover(203-221), Figure 4A] was still able to suppress photoadduct formation (note residual signal for free CaM in lane 12 in Figure 4B and corresponding mass spectrum in Figure 4C), at least when applied in 50-fold molar excess, indicating that this peptide retained some residual affinity to CaM. As this resembles the situation seen with the CaM-binding motif of bMunc13-2 (Lipstein et al., 2012), we decided to follow a similar strategy as we did previously for eliminating the CaM-binding of that particular Munc13 isoform. In addition to the exchange of the hydrophobic anchor residue, we also targeted the basic patch at the opposite site of the presumed alpha helix by replacing three Lys with Glu residues (K207E, K215E, and K219E; Supplementary Figure S4). This peptide, referred to as R/E-Mover (Figure 4A), was unable to suppress photoadduct formation (note absent signal for free CaM in lane 14 in Figure 4B and corresponding mass spectrum in Figure 4C), indicating that the mutant form does not bind to CaM anymore. Taken together, PAL-based competition assays revealed that a peptide comprising the predicted CaM-binding sequence of rat Mover (amino acids 203-221) indeed binds to CaM in a strictly Ca^2+^-dependent manner. In comparison to the WT sequence, CaM binding is impaired in the F206R variant, and can be completely abolished when the mutations K207E, K215E and K219E are introduced in addition.

**Figure 5:**
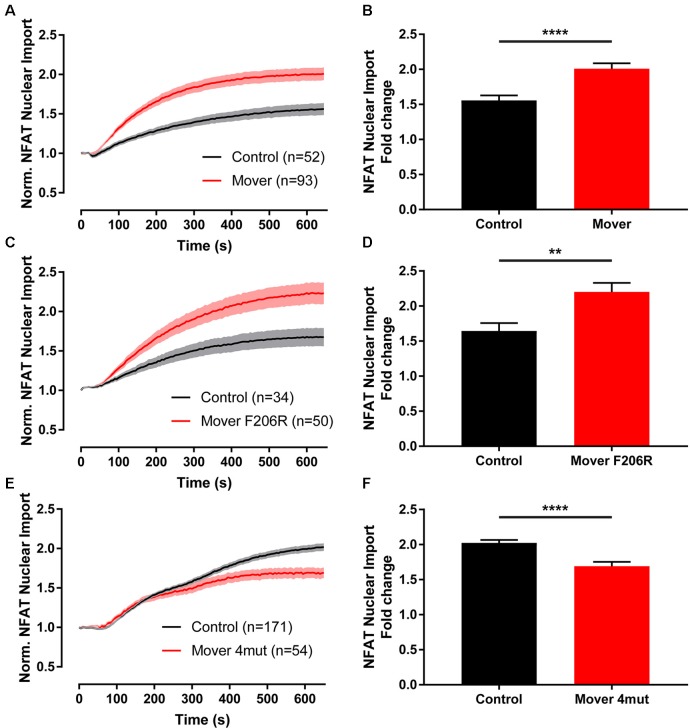
NFAT1-GFP translocation is enhanced by overexpression of Mover in HeLa cells. **(A)** The dynamics of nuclear NFAT1-GFP fluorescence was imaged upon histamine stimulation of live HeLa cells expressing either recombinant full-length Mover (red color) or DsRed as a control (black color). Expressing Mover increases the histamine-induced accumulation of NFAT1-GFP in the nucleus. **(B)** Quantification of the nuclear NFAT1-GFP fluorescence as fold change at the end of the experiment vs. the beginning (*p* < 0.0001). **(C,D)** Full-length Mover carrying the F206R point mutation still increases histamine-induced NFAT1-GFP nuclear translocation (*p* = 0.004). **(E,F)** The F206R/K207E/K215E/K219E quadruple mutant, called Mover 4mut, fails to increase the histamine induced NFAT1-GFP nuclear translocation (*p* < 0.0001). Two independent experiments were performed, Mann–Whitney test was used, *n* is indicated in the panels. **p < 0.01,****p < 0.0001.

Experiments using knockdown of Mover at the calyx of Held had raised the possibility that Mover and CaM may interact functionally, either by Mover affecting CaM or vice versa (Korber et al., 2015). To test whether Mover can affect a certain CaM signaling pathway in living cells we applied an assay that determines the Ca/CaM-dependent translocation of the transcription factor NFAT1 from the cytosol to the nucleus (Zhang et al., 2019). In this assay, application of histamine, an activator of the store operated Ca^2+^ entry pathway, induces an increase in cytoplasmic calcium levels. Calcium-laden CaM then binds to and activates the phosphatase calcineurin. Calcineurin dephosphorylates the nuclear localization signal of NFAT1, thus triggering the translocation of NFAT1 to the nucleus. A histamine-induced increase in nuclear GFP-NFAT1 is thus a readout for active CaM signaling. If Mover stimulates or inhibits CaM action, overexpression of Mover should lead to a change in NFAT1-GFP fluorescence in this assay. Likewise, if Mover acts downstream of CaM in this assay, overexpression of Mover should affect nuclear NFAT1-GFP fluorescence. To conduct the assay, we generated a new construct encoding untagged rat Mover. To visualize the transfected cells, the red fluorescent protein mKate is translated from an internal ribosomal entry site (IRES) in this construct. We co-transfected HeLa cells either with GFP-tagged NFAT1 (NFAT1-GFP) and Mover-IRES-mKate or, as a control, with NFAT1-GFP and dsRed. We then monitored the nuclear NFAT1-GFP signal over time in live cells. Histamine application caused an increase in the nuclear NFAT1-GFP signal, which was more rapid and more extensive in the Mover-IRES-mKate over-expressing cells (Figures 5A,B, *p* < 0.0001 Mann–Whitney test). Thus, recombinant Mover increased Ca/CaM-induced NFAT1 translocation. A point mutated variant of full-length Mover harboring the F206R mutation still increased Ca/CaM-induced NFAT1 translocation (Figures 5C,D, *p* = 0.004 Mann–Whitney test). This is consistent with our PAL-based competition assays, which showed that the F206R mutated peptide still bound to CaM. In contrast, a variant of full-length Mover harboring the four mutations that disrupted CaM binding in our competition assay failed to increase the nuclear NFAT1 signal (Figures 5E,F, *p* < 0.0001 Mann–Whitney test). While the action of WT Mover and the F206R mutated construct were visible within seconds of histamine application, the quadruple mutant failed to have any effect over the first 200 s of the experiment. Later in the experiment, this mutant reduced NFAT translocation to below control levels, suggesting that over the course of the experiment it may have started to develop some dominant-negative or generally deleterious effects. Overall, the data reveal that Mover increases Ca/CaM-induced NFAT1 translocation, indicating that it boosts CaM signaling in this assay.

**Figure 6:**
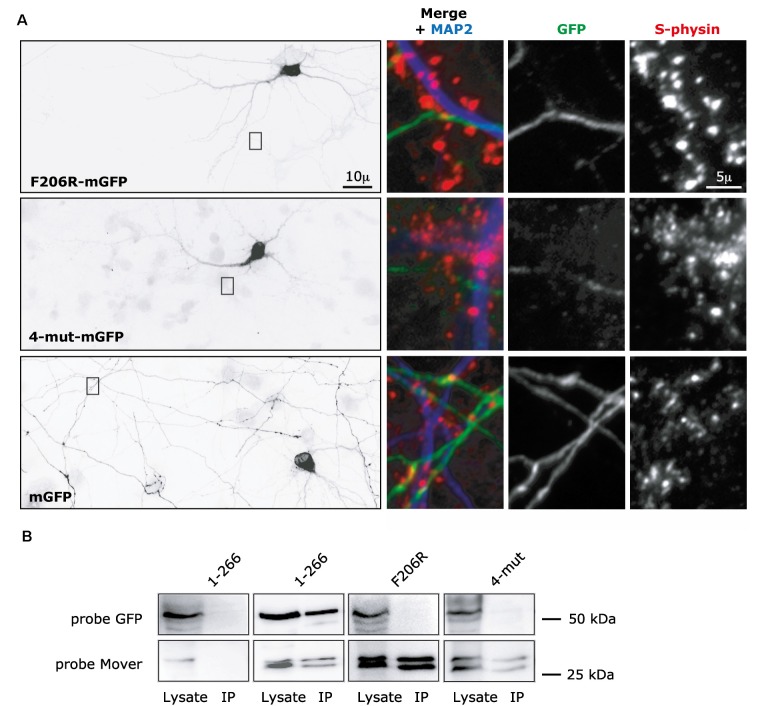
Targeting and self-interaction properties of Mover constructs containing mutations in the CaM binding domain. **(A)** F206R-mGFP, corresponding to full-length Mover carrying one point mutation, and 4-mut-mGFP, corresponding to full-length Mover carrying four point mutations (F206R, K207E, K215E and K219E) are diffusely distributed in rat cultured hippocampal neurons. Their distribution resembled that of soluble mGFP alone. The overviews (left) show inverted gray level epifluorescence images of GFP-immunofluorescence. The zooms on the right represent triple-fluorescence images of the boxes, showing that axonal areas (MAP2 negative processes) of the transfected neurons do not contain local accumulation of the constructs even where the axon contacts a dendrite (MAP2 positive process), i.e., at a location where synapses can be formed. Synaptophysin (S-physin) is a marker for presynaptic SV clusters. The distribution of each construct was analyzed in more than 30 neurons from three independent experiments. **(B)** Full-length Mover (1-266) co-immunoprecipitates with Mover-myc in transfected HEK293 cells, but the F206R mutant and the 4-mut mutant do not, indicating that these point mutations disrupt homomeric interaction of Mover. The data represent two independent experiments, i.e., two HEK293 cell transfections followed by lysis and co-immunoprecipitation.

Next, we tested how the CaM binding region affects presynaptic targeting and self-interaction of Mover. We generated two new versions of full-length Mover, each with a C-terminal mGFP-tag: a point-mutated version carrying the F206R mutation, called F206R-mGFP, and a version carrying all four mutations (F206R, K207E, K215E and K 219E), called 4-mut-mGFP. Strikingly, both constructs failed to produce any punctate fluorescence. Instead, they were diffusely distributed in the transfected neurons (Figure 6A). We verified the absence of presynaptic targeting by triple-staining the neurons using anti-MAP2 to detect dendrites, anti-Synaptophysin to detect presynaptic SV clusters, and anti-GFP antibody to detect even very low level GFP expression. We specifically searched for areas where an axon contacted a dendrite and where Synaptophysin staining indicated the presence of synapses. Even at such selected areas, the constructs were diffusely distributed within the axon, indicating that even where these axons can clearly make synapses there was no accumulation of the construct (Figure 6A). Comparing the distribution of the constructs to the distribution of mGFP alone further corroborated that the constructs were as diffusely distributed as soluble mGFP (Figure 6A). In low-magnification views, it even appeared as if the constructs were partially excluded from axons, but this may be due to lower expression levels and thus lower signal intensity compared to mGFP. Interestingly, these two constructs also failed to co-immunoprecitipate with Mover-myc (Figure 6B). Together, these data indicate that the two constructs fail to self-interact and fail to accumulate at presynaptic sites. Strikingly, only a specific point mutation (F206) is required to abolish self-interaction and targeting.

**Figure 7:**
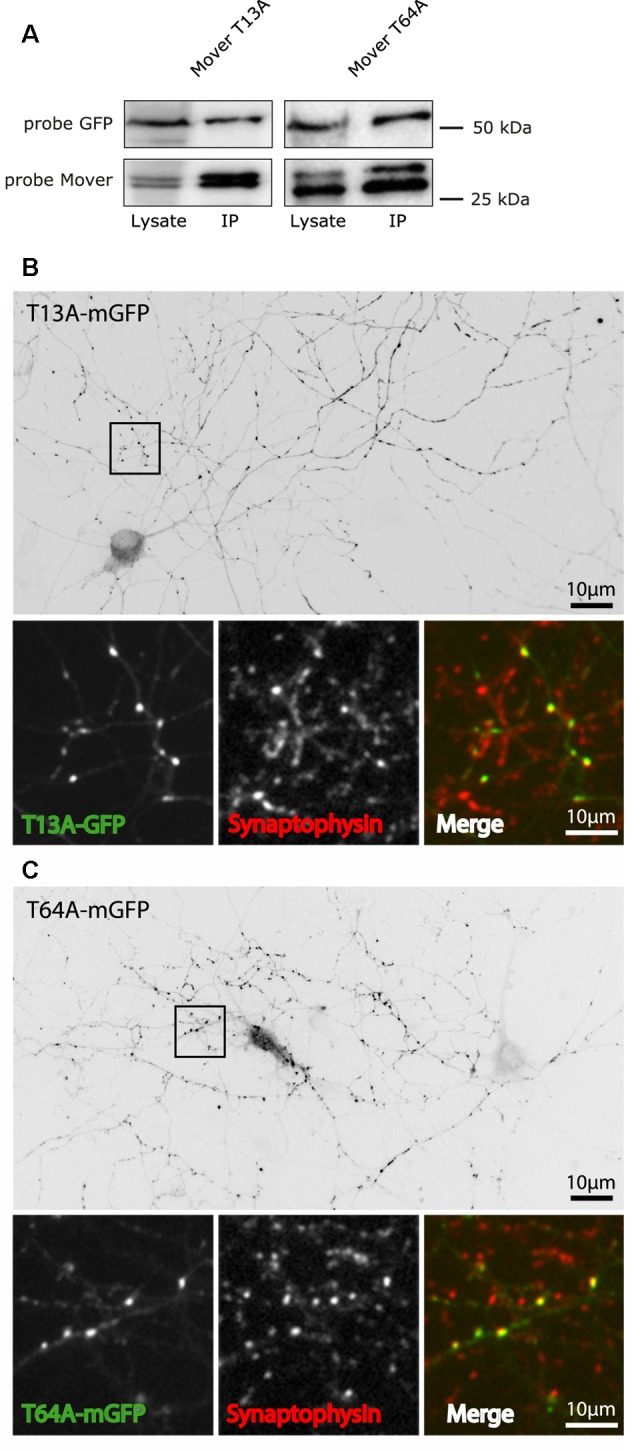
Targeting and self-interaction properties of phospho-deficient mutants of Mover. **(A)** The T13A mutant and the T64A mutant of Mover co-immunoprecipitates with Mover-myc in transfected HEK293 cells, indicating that they undergo homomeric interaction. The data represent two independent experiments. **(B,C)** The two phospho-deficient mutants are targeted to presynaptic sites in rat cultured hippocampal neurons. This is indicated both by the punctate staining pattern (inverted gray level images) and the colocalization with the synapse marker Synaptophysin (small panels, representing zoomed version of the boxes). The distribution of each construct was analyzed in more than 15 neurons from three independent experiments.

In addition to self-interaction and CaM binding, phosphorylation may contribute to the properties of Mover. Mover has a functional phosphorylation site at T13, and a predicted phosphorylation site at T64. To test their implication in targeting and self-interaction, we generated a phospho-deficient mutant for each of these sites, termed T13A-mGFP and T64A-mGFP, respectively. Both mutants co-immunoprecipitated with Mover-myc (Figure 7A) and accumulated at presynaptic sites in transfected cultured neurons (Figures 7B,C). Since these mutants are capable of self-interaction they might piggy-back to synapses on endogenous Mover, without actually having intrinsic targeting capacity. Thus, we expressed these mutants in neuronal cultures obtained from a global Mover knockout line that we generated in the lab (Supplementary Figure S5).

**Figure 8:**
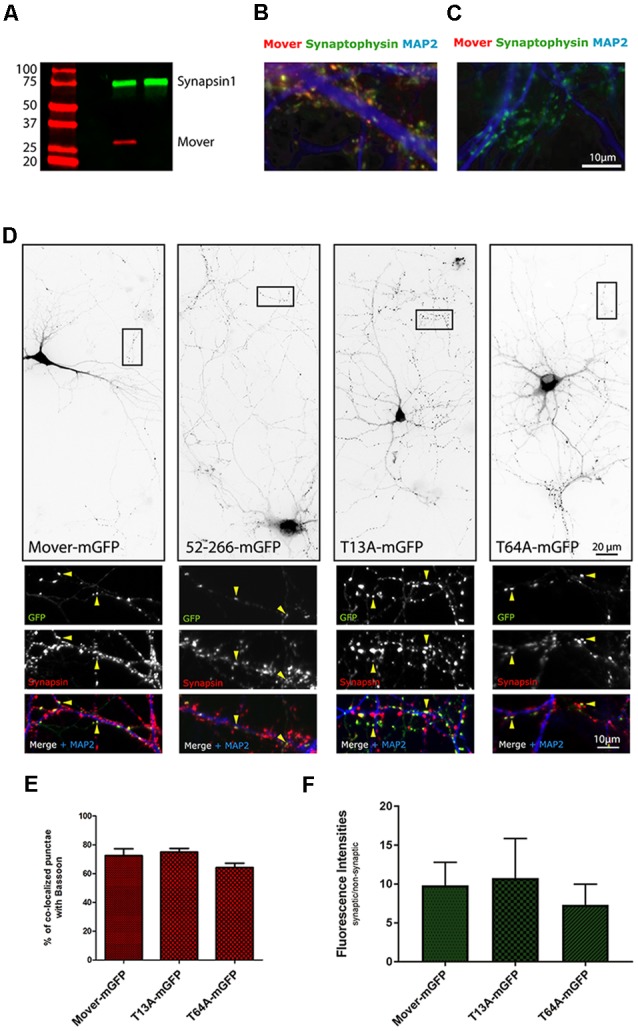
Targeting of Mover constructs in Mover knockout neurons. **(A)** Western blot demonstrating the absence of Mover in a brain homogenate from Mover knockout mice. **(B,C)** Immunofluorescence of cultured hippocampal neurons from WT mice **(B)** and Mover knockout mice **(C)** indicating the absence of Mover immunofluorescence in the knockout cultures. **(D)** Inverted gray level images showing the distribution of the indicated constructs in transfected Mover knockout cultures. All constructs produce a punctate staining characteristic of presynaptic targeting. The small panels represent zooms of the boxes. Arrowheads show examples of punctate colocalization of the constructs with the synapse marker Synapsin. **(E)** Quantification of colocalization with the synapse marker Bassoon. The number of punctate GFP-signals colocalizing with Bassoon is shown as percentage of the total number of punctate GFP-signals (*n* = 18 regions of interest; *N* = 6 independent experiments; student’s *t*-test). **(F)** Enrichment of the constructs at synapses. The average fluorescence intensity of punctate GFP-signals colocalizing with a synapse marker and the average fluorescence intensity of nearby diffuse GFP-signals (i.e., non-synaptic signals) was determined. The ratio of synaptic vs. non-synaptic fluorescence signals is shown (*n* = 18 regions of interest, including 540 synapses total; *N* = 6 independent experiments; student’s *t*-test).

Western blotting confirmed the absence of Mover in brain homogenate (Figure 8A), and immunofluorescence confirmed the absence of Mover in cultured neurons (Figures 8B,C). The basic parameters of synaptic transmission were not altered in knockout neurons (Supplementary Figures S6, S7). In dissociated hippocampal neurons, spontaneous transmission was unaffected, as evidenced by normal mEPSC amplitude, frequency, rise time and the time constant of decay (Supplementary Figure S6), indicating that the pre- and post-synaptic apparatus was unaffected. In microisland cultures, where autaptic connections can be analyzed, key parameters of basal synaptic transmission and of synaptic plasticity were unaffected. These included the readily releasable pool of SVs, vesicular release probability, mEPSC frequency and amplitude, as well as the paired-pulse ratio, steady-state responses and recovery after trains of stimulation (Supplementary Figure S7). This emphasizes the notion that Mover may be particularly important at specialized synapses such as the Calyx of Held (Korber et al., 2015). They also indicate that Mover KO cultures are vital and can be used to study the targeting behavior of Mover constructs.

When expressed in these cultures, Mover-mGFP and 52-266-mGFP produced the expected punctate staining pattern, and the puncta colocalized with the presynaptic marker Synapsin (Figure 8D). This confirms that these two constructs have intrinsic targeting capacity. Likewise, the two phospho-mutants produced a punctate staining pattern with the puncta co-localizing with the presynaptic markers Synapsin (Figure 8D) and Bassoon (Supplementary Figure S8). Quantification corroborated this observation: Mover-mGFP, T13A-mGFP and T64A-mGFP showed the same percentage of co-localization with Bassoon (Figure 8E; Colocalization with Bassoon: 72.39 ± 4.82 for Mover-mGFP; 74.93 ± 2.62 for T13A-mGFP; 64.08 ± 3.13 for T64A-mGFP; *p* = 0.65 for Mover-mGFP/T13A-mGFP; *p* = 0.16 for Mover-mGFP/T64A-mGFP; student’s *t*-test; *n* = 18; *N* = 6). To further corroborate these findings we quantified the average synaptic enrichment of these constructs, by determining the ratio of synaptic GFP-fluorescence (i.e., GFP-signal co-localizing with Bassoon) compared to non-synaptic GFP-fluorescence (i.e., GFP-signal next to nearby synapses, but not co-localizing with Bassoon). The T13A phospho-mutant showed similar synaptic enrichment compared to Mover-mGFP, while T64A-mGFP showed lower enrichment (Figure 8F; synaptic enrichment: 9.84 ± 0.70 for Mover-mGFP; 10.78 ± 1.20 for T13A-mGFP; 7.34 ± 0.62 for T64A-mGFP; *p* = 0.50 for Mover-mGFP/T13A-mGFP; *p* = 0.01 for Mover-mGFP/T64A-mGFP; student’s *t*-test; *n* = 18; *N* = 6). These data indicate that the mutant constructs 52-266-mGFP, T13A-mGFP and T64A-mGFP each have intrinsic targeting capacity. They also reveal that phosphorylation at either T13 or T64 is not required for the targeting of Mover to synapses, but that T64 may promote the accumulation of Mover at each synaptic site.

## Discussion

Mover is a peripheral membrane protein of SVs with a tendency to undergo homomeric interaction (Ahmed et al., 2013). Because Mover has no functionally characterized homologs in invertebrates, it is difficult to pinpoint potentially important domains and regions based on their evolutionary conservation. To characterize its molecular properties nonetheless, we have now performed a deletion/mutation analysis on recombinant rat Mover, and investigated three of its most prominent features: its accumulation in presynaptic terminals, its tendency to undergo homomeric interaction, and the presence of two predicted functional domains, including an hSac2 homology domain and a predicted CaM binding region.

Our primary findings are: first, that regions required for targeting and homomeric interaction are distributed over virtually the entire protein, indicating that there is no single functional domain mediating these activities, and that the overall structure of the entire protein has to be intact to mediate these properties—our FRET analysis confirmed that recombinant Mover undergoes homomeric interaction in neurons; second, mutations that abolished homomeric interaction also abolished presynaptic targeting. Phosphorylation at T13 or T64 is not required for any of these properties. However, a point mutation exchanging phenylalanine at position 206 for arginine (F206R) was sufficient to abolish both presynaptic targeting and homomeric interaction, highlighting the particular importance of this region; and third, F206 is part of a functional CaM binding motif that resembles the motif found in the priming protein bMunc13-2; fourth, Mover activates CaM signaling upon heterologous expression, revealing the potency of Mover for regulating CaM action.

### Deletion Analysis: Premise and Outcome

Mover contains an hSac2 homology domain spanning amino acids 53 through 163. This domain exists in the Mover paralog TPRG, a protein that has 44% identity with Mover and may have arisen in vertebrates as part of a gene duplication event (Antonini et al., 2008). It also exists in a protein called Sac2, a phosphatidylinositol phosphatase that occurs both in vertebrates and invertebrates (Hsu and Mao, 2013). We found that the GFP-tagged version of the hSac2 domain of Mover was diffusely distributed in transfected neurons, indicating that it does not contain information sufficient to allow for presynaptic accumulation, even though it covers more than half of the sequence of Mover that does contain targeting information, i.e., amino acids 52-266. The sequences flanking the hSac2 domain upstream or downstream are not sufficient either, as evidenced by the diffuse distribution of all of our deletion constructs except 52-266-mGFP. For example, even removing only 13 amino acids from the C-terminus of 52-266-mGFP abolished presynaptic targeting. Thus, the essential sequences are distributed virtually across the entire protein, but none of them alone is sufficient.

One of the diffusely distributed deletion constructs, called Δ93-151, lacks a central portion of the hSac2 domain and mimics a putative splice isoform of Mover lacking exon 2 that is predicted by database entries. Thus, if such an isoform exists it can be expected to be diffusely distributed in the neuronal cytosol, rather than acting primarily at synapses. Only a short N-terminal region is dispensable for targeting. This region, comprised of amino acids 1–51, is predicted to be structurally disordered. It is also the only variable region among the vertebrate Mover orthologs (Kremer et al., 2007; Antonini et al., 2008). It may lack functional importance, or it may be adapted for special functions in distinct vertebrate species.

Mover is phosphorylated at T13 in this variable region (Munton et al., 2007; Ahmed et al., 2013). We find here that preventing phosphorylation at T13 did not impair presynaptic targeting. We can rule out that targeting occurred by piggy-backing on endogenous Mover because it even occurred in cultures from Mover knockout mice. Likewise, preventing phosphorylation at T64, a predicted phosphorylation site, did not impair targeting. We had previously shown that dephosphorylating SV fractions lead to the detachment of endogenous Mover from SVs (Ahmed et al., 2013). This could be either because a site in Mover becomes dephosphorylated during the phosphatase treatment, or because an unknown protein required for SV attachment of Mover becomes dephosphorylated. Because T13 is known to be phosphorylated, and because the predicted phosphorylation site T64 is part of the hSac2 domain, we tested here whether one of these two sites is required for the accumulation of Mover in presynaptic terminals. Our phospho-deficient mutants show that phosphorylation at T13 or T64 is not required, as evidenced by the same percentage of colocalization with synaptic markers of the T13A and T64A mutants compared to the WT protein. The T64A mutant showed a trend towards reduced enrichment at synapses, but this trend was only significant in one of two statistical tests that we applied. Phosphorylation at T64 is thus not required for presynaptic targeting, but it may have a subtle contribution to the amount of Mover accumulating in presynaptic terminals.

### Dimerization and Targeting

We had previously shown that presynaptic targeting of Mover requires amino acids 52-91, i.e., an N-terminal portion of the hSac2 domain. Here we found that it requires, in addition, a central portion of the hSac2 domain (amino acids 93-151), as well as F206, and the last 13 amino acids of Mover. Interestingly, the constructs that fail to accumulate at synapse also fail to bind to full-length Mover, and the constructs that do accumulate at synapses are also capable of binding to full-length Mover. This includes both phospho-mutants, which are capable of both presynaptic targeting and binding to full-length Mover, consistent with the notion that homomeric interaction and presynaptic targeting of Mover are connected. These observations also suggest that endogenous Mover exists as a dimer or oligomer. This idea is corroborated by our FRET analysis, showing that recombinant Mover produces FRET in transfected neurons. FRET occurred when both fluorescent tags were located at the N-terminus of Mover, but not when one was located at the N-terminus and the other one at the C-terminus. This is consistent with the assumption that Mover occurs as a dimer with the monomers oriented in parallel, but we cannot exclude the possibility that higher-order oligomers with a more complex arrangement of monomers exist.

Dimerization is required for the synaptic accumulation of other presynaptic proteins as well, including, e.g., Bassoon, Synapsins and GAD65 (Gitler et al., 2004; Kanaani et al., 2010; Maas et al., 2012). The regions required for dimerization of Synapsins are spread out over several domains, reminiscent of what we observe for Mover. The reason why dimerization is required for presynaptic targeting in these proteins is unknown, and consensus sequences mediating presynaptic targeting have not been identified. It has been suggested that SVs might recruit proteins to synapses (Denker et al., 2011; Rizzoli, 2014). Perhaps proteins that are capable of dimerizing and binding to SVs at the same time can travel with SV clusters most effectively: one monomer may recruit the other monomer, and by binding to two vesicles the dimer may contribute to vesicle clustering, which in turn would increase protein recruitment.

While our deletion analysis primarily shows that regions required for targeting and homomeric interaction are spread out across large portions of Mover, it also adds some novel insights into the properties of the individual domains. First, the Mover deletion construct lacking amino acids 1-52 binds full-length Mover and targets to synapses in cultured rat neurons, as shown here and previously (Ahmed et al., 2013). So far, we had not been able to exclude the possibility that it interacts with endogenous Mover and accumulates at synapses by “piggy-backing”. Here, we found that it also accumulates at synapses in cultured neurons from mice lacking Mover, indicating that it indeed has inherent targeting capacity. Second, the hSac2 homology domain of Mover is required for homomeric interaction but not sufficient for it. This is consistent with studies performed on the hSac2 domain of the eponymous Sac2 protein (Hsu and Mao, 2013; Hsu et al., 2015). Sac2 is a 128 kDa protein capable of dimerizing (Hsu et al., 2015). It contains a Sac1 domain, which carries phosphatidylinositol phosphatase activity, and the hSac2 domain, whose function is unclear. Deleting the hSac2 domain from Sac2 prevents the dimerization of the deletion construct with full-length Sac2 (Hsu et al., 2015), indicating that, like the hSac2 domain of Mover, this domain is required for homomeric interaction. Whether the hSac2 domain of Sac2 alone is sufficient for dimerization was not tested. Our data show that the hSac2 domain is not sufficient for homomeric interaction of Mover.

### The Predicted CaM Binding Sequence Is Functional and Relevant for the Structural Integrity of Mover

We had previously shown that recombinant CaM pulls down endogenous Mover from rat brain homogenate, and that recombinant CaM binds recombinant Mover directly (Korber et al., 2015). However, which part of Mover binds to CaM remained unknown. Here, we identify, using bioinformatics tools and a PAL assay (Dimova et al., 2006; Lipstein et al., 2012) the amino acid stretch 203-221 of Mover as the functional CaM binding site. This was revealed by gel electrophoresis showing the formation of covalent higher molecular weight adducts of CaM in the presence of the photoreactive Mover(203-221) peptide, and was confirmed by MS. MS also revealed that in addition to a 1:1 Mover-CaM-photoadduct (consisting of a 17 kDa CaM component and a 2 kDa peptide component), a ~21 kDa adduct was apparent, which would be consistent with a 2:1 stoichiometry in addition to the 1:1 stoichiometry (17 kDa CaM, two times 2 kDa peptide). However, such higher stoichiometries often appear as by-products in this assay when minimal CaM-binding peptides are incubated under conditions of high Ca^2+^ concentrations and subjected to long irradiation times (Dimova et al., 2006). Based on our experience with CaM-binding peptides derived from Munc13 s (Junge et al., 2004; Dimova et al., 2006, 2009; Lipstein et al., 2012), we consider it more likely that the 1:1 Mover/CaM complex is the biologically relevant species, but cannot fully exclude the existence of 2:1 stoichiometry at the cellular level.

CaM binding sequences are characterized by a net positive charge and the presence of hydrophobic residues with a characteristic spacing (Lipstein et al., 2017). For example, the active zone protein Munc13-1 and its highly homologous isoform ubMunc13-2 have a 1-5-8-26 spacing of the positions of these hydrophobic residues within the CaM binding sequence, while the brain-specific isoform bMunc13-2 has a 1-5-10 spacing, which may be related to its lower level of homology in the N-terminal part (Lipstein et al., 2012). Interestingly, CaM binding can be abolished by mutating only one amino acid in the CaM binding sequence of Munc13-1 and ubMunc13-2, i.e., the tryptophan in position 1 of the binding sequence (Junge et al., 2004; Lipstein et al., 2012, 2013). In contrast, mutating the equivalent amino acid in the isoform bMunc13-2, i.e., exchanging a phenylalanine for arginine, only reduced CaM binding. Here, the additional exchange of three positively charged amino acids of the putative CaM-binding alpha-helix was required to abolish CaM binding of bMunc13-2 (Lipstein et al., 2012), possibly indicating that the hydrophobic contact sites crucial for CaM binding are not yet fully understood in this Munc13 isoform. With respect to the full-length sequence of rat bMunc13-2, the four mutations required to abolish CaM binding are F723R, K724E, R728E, and R731. We mimicked these mutations by introducing F206R, K207E, K215E, and K219E (see helical wheel projections in Supplementary Figure S4), and this combination did abolish CaM binding in the PAL assay, whereas a peptide merely containing the F206R mutation still retained some affinity to CaM. Thus, the CaM binding site of Mover shares more similarity with that of bMunc13-2 than with that of Munc13-1/ubMunc13-2. The significance of this similarity is unknown. One possible scenario is that at the synapse Mover may compete with bMunc13-2 for CaM binding. Because Mover is heterogeneously expressed at subsets of synapses (Wallrafen and Dresbach, 2018) it may regulate bMunc13-2 dependent vesicle priming at these synapses.

Remarkably, the CaM binding region of Mover is apparently crucial for the overall structure of Mover. Introducing the F206R point mutation into Mover was sufficient to abolish both homomeric interaction with WT Mover and presynaptic targeting: the point-mutated construct was diffusely distributed in transfected neurons, failed to accumulate at synapses and failed to bind to WT Mover. The complete loss of these properties by one point-mutation is striking. In contrast, point-mutating the CaM binding region of the SV priming protein Munc13-1 abolished CaM binding but did not impair its targeting to presynaptic terminals (Lipstein et al., 2013). The CaM binding region of Mover thus has importance for the overall structure of Mover, and by extension for its dimerization and presynaptic targeting.

### Enhancing CaM Signaling Is a Novel Feature of Mover

What is the role of Mover in CaM signaling? Knockdown of Mover at the rat Calyx of Held synapse increased the probability of transmitter release and the sensitivity of the transmitter release machinery for calcium. Because CaM interacts with numerous proteins involved in regulating neurotransmitter release (Chin and Means, 2000; Junge et al., 2004; Dick et al., 2008; Di Giovanni et al., 2010; Lipstein et al., 2017), we had speculated that the effect of the knockdown could result, at least in part, from a reduced interaction of Mover with CaM (Korber et al., 2015). However, testing this prediction is impossible because, as we find here, mutating the CaM binding region prevents the targeting of Mover to synapses. Thus, rescue experiments with a CaM binding deficient variant of Mover are not feasible. To still address whether or not Mover is involved in CaM signaling, we used a reduced system where CaM signaling can be read out as the Ca/CaM/Calcineurin dependent translocation of the transcription factor NFAT1 from the cytosol to the nucleus of cultured HeLa cells. In this pathway, a rise in intracellular calcium activates CaM, which in turn binds and activates the CaM-dependent phosphatase Calcineurin. Activated Calcineurin dephosphorylates the nuclear localization signal of the transcription factor NFAT, which leads to the translocation of NFAT from the cytoplasm to the nucleus (Rao et al., 1997; Crabtree and Olson, 2002). We overexpressed Mover, using histamine induced GFP-NFAT1 translocation as a gain-of-function assay for the role of Mover in this pathway. This approach yielded several intriguing results. First, it revealed that overexpressing Mover boosts the Ca/CaM/Calcineurin pathway in this system. This is important because it had been unclear whether or not Mover interferes with CaM-related pathways at all, and whether it enhances or inhibits CaM signaling. The experiment reveals that Mover enhances this particular CaM signaling pathway. Second, binding of Mover to CaM is required for this enhancement. The fact that the F206R mutant of Mover still increased NFAT1 translocation is consistent with the results from our peptide competition assay, which showed that the F206 mutant Mover peptide still binds to CaM. Likewise, the lack of an activating effect of the quadruple mutant is consistent with the quadruple mutant peptide not binding to CaM in the competition assay. While the quadruple mutant was completely without effect over the first 200 s of the experiment, it developed an inhibitory effect later on. Several explanations could account for this: (i) the construct might have a generally deleterious effect on the cells that develop over time; and (ii) Mover may have as yet unidentified binding partners whose function is perturbed by the quadruple mutant over the course of the experiment. This latter notion raises an interesting possibility: Mover may bind Calcineurin, and activation of Mover by CaM may increase the phosphatase activity of Calcineurin. In this scenario, a CaM/Mover complex would provide additional activation to Calcineurin, on top of Calcineurin activation *via* CaM directly. The CaM-binding deficient Mover mutant would then bind to Calcineurin but fail to activate it, leading to a reduction in the activation of the Calcineurin pathway, as observed in the NFAT assay. In any case, our gain-of-function assay provides the first demonstration that Mover can functionally interact with CaM, and reveals that Mover can positively modulate the CaM/Calcineurin/NFAT1-signaling pathway.

Overall, it is an intriguing possibility that a vertebrate-specific protein may add versatility to the conserved CaM signaling pathways. By extension, in presynaptic terminals Mover may connect the specialized components of the transmitter release machinery, in particular Bassoon, to conserved components such as CaM and Munc13s. The structural properties of Mover, where homomeric interaction and presynaptic accumulation appear to be connected, may direct its potency to modulate CaM signaling to sites of transmitter release.

## Data Availability Statement

The datasets generated for this study are available on request to the corresponding author.

## Ethics Statement

The animal study was reviewed and approved by State Government of Lower Saxony, Germany.

## Author Contributions

AA performed targeting and immunoprecipitation experiments, analyzed the data, and performed the majority of the work generating the knockout mouse line. XZ performed the NFAT assay and analyzed the data. JV performed electrophysiology on mass cultures, analyzed the data, and contributed to statistical analyses and study design. DN performed electrophysiology on autaptic cultures and analyzed the data. J-SR supervised the autapse experiments. RE performed and analyzed the FRET assay. KR contributed to creating the knockout mouse line. FW supervised the FRET experiments. TL generated and analyzed the photoaffinity labeling-based peptide competition data. OJ designed and performed the photoaffinity labeling assay. IB designed and supervised NFAT assay. TD designed the project, analyzed the data and wrote the manuscript.

## Conflict of Interest

The authors declare that the research was conducted in the absence of any commercial or financial relationships that could be construed as a potential conflict of interest.

## Supplementary Material

FIGURE S1 Triple immunostaining of synaptically targeted constructs for quantitative analysis. **(A–C)** Fluorescence microscopy of GFP-tagged rat Mover constructs and GFP tagged VAMP (a marker of synaptic vesicles) in DIV14 rat hippocampal neurons. The inverted gray level images represent GFP fluorescence detected with a 20× objective. The cultures were triple stained for GFP, the synaptic vesicle marker Synaptophysin and the dendrite marker MAP2. A 40× objective was used to record triple fluorescence images. Examples of such triple fluorescence images are shown in the small panels, and the boxes indicate where these examples are located in the 20× images. For quantitative analysis of the synaptic accumulation of the recombinant proteins the average fluorescent intensity of a synaptically localized punctate GFP-signal was divided by the average GFP-fluorescence intensity of a nearby axonal region. Punctate GFP-signals were defined as synaptic if they colocalized with the synapse marker Synaptophysin at a dendrite, identified by MAP2. **(D)** The ratio of synaptic vs. non-synaptic GFP-fluorescence, representing the enrichment of the protein at synapses compared to non-synaptic sites, of Mover-mGFP and 52-266-mGFP was similar to the enrichment of the presynaptic marker GFP-VAMP (*n* = 6 regions of interest, each representing 10 synaptic and 10 non-synaptic sites. *N* = 2 independent experiments. One-way ANOVA test; *p* = 0.32).

FIGURE S2 Triple immunostaining of homogeneously distributed constructs. **(A–C)** Fluorescence microscopy of GFP-tagged rat Mover constructs in DIV14 rat hippocampal neurons, indicating that these recombinant proteins are homogeneously distributed. The areas indicated by the boxes are zoomed in the small panels. Arrowheads indicate examples of axons, identified as GFP-positive and MAP2-negative processes. The constructs are homogeneously distributed in the axons, even when the axon contacts a dendrite. The punctate Synaptophysin staining indicates that synapses do exist in the cultures. The distribution of each construct was determined in more than 30 neurons on a total of six coverslips from three independent experiments (the same neurons and experiments as in Figure 1).

FIGURE S3 Characterization of recombinant Mover constructs expressed in HEK293 cells. Lysates from untransfected HEK293 cells and HEK293 cells expressing one of the Mover constructs were analyzed by western blotting. **(A)** Western Blot of lysates from cells expressing the indicated constructs, probed with rabbit anti-GFP. Based on their amino acid sequence the following molecular weights of the GFP-fusion proteins are expected: 57 kDa for 1–266, 52 kDa for 52–266, 50 kDa for 53–253, 52 kDa for Δ93–151, and 40 kDa for 53–163. The observed bands correspond to these molecular weights. In addition, 1–266 and, to a smaller extent, 52–266 produce smaller molecular weight bands, probably representing proteolytic degradation products. **(B)** Western Blot of lysates from cells expressing the indicated constructs, probed with rabbit anti-Mover. All constructs are detected by the Mover antibody and display the predicted molecular weights. In addition, the Mover antibody detects a ca. 32 kDa band in untranstected cells, which may correspond to the human Mover variant FAM79A. Mover-myc runs appears as double band. Both bands have higher molecular weight than untagged recombinant Mover. The data represent three independent experiments, i.e., three HEK293 cell transfections followed by lysis.

FIGURE S4 Helical wheel projections of CaM-binding peptides derived from Mover **(A)** and bMunc13-2 **(B)**. For the generation of CaM-insensitive Mover, a similar strategy was applied as previously established in bMunc13-2. In addition to the replacement of the hydrophobic anchor residue F4 in Mover(203-221) by a charged Arg residue, the Lys residues forming the basic patch at the opposite site of the presumed alpha-helix (K5, K13, K17) were replaced by acidic Glu residues. As Lys and Glu have similar propensities to form an alpha helix, the charge of this helix patch was switched without changing the overall secondary structure.

FIGURE S5 Knockout strategy. Schematic representation of the knockout strategy **(A)**. The entire Mover gene including exons and introns spans 3616 base pairs. Blue triangles represent loxP sites. Green triangles represent FRT sites used to remove the Neo cassette *via* FLP recombinase. The 5’ loxP site is located upstream of the translation start site in the 5’ UTR region of exon 1. Cre mediated excision removes exons 1 through 3 and part of the downstream intron.Three primers (P4, E3001 and E4001) were used for genotyping **(B)**. A PCR reaction including all three primers produces a 867 bp product on wildtype DNA (generated by P4 and P3), a 697 bp product on knockout DNA (generated by P4 and E4001), and both products on DNA from heterozygous animals.

FIGURE S6 Spontaneous transmission in cultured neurons is unchanged in the absence of Mover. Whole cell recordings of miniature EPSCs in hippocampal dissociated cultures measured in the presence of 1 μM TTX. **(A,B)** Representative current traces of mEPSC recordings in WT (*n* = 31 cells) and Mover KO cultured neurons (*n* = 28 cells). **(C)** mEPSC amplitude, **(D)** frequency, **(E)** 10–90 rise time, and **(F)** time constant of decay are unchanged. Error bars represent SEM.

FIGURE S7 Synaptic transmission is not altered in cultured autaptic hippocampal neurons from Mover KO mice. **(A)** Representative traces of AP-evoked EPSCs (i) spontaneously occurring mEPSCs (ii) and sucrose-evoked EPSCs in cultured autaptic neurons. **(B)** Mean amplitude of AP-evoked EPSCs (*n* = 31 and 32 cells). **(C)** RRP size measured in response to 0.5 M sucrose (*n* = 30 and 32 cells) **(D)** Mean vesicular release probability calculated by dividing the charge of AP-evoked EPSCs by the charge of sucrose evoked responses (*n* = 30 and 32 cells). **(E)** and **(F)** Mean amplitude and frequency of mEPSCs measured in presence of 300 nM TTX for 100s. (*n* = 29 and 25 cells). **(G)** Averaged EPSC responses during a 40 Hz AP train (*n* = 9 cells each) **(H)** and **(I)** Paired-pulse ratio and steady-state EPSC responses during trains of APs at indicated frequencies (*n* = 29 and 30 cells for 10 Hz, *n* = 9 cells each for 40 Hz stimulation). **(J)** Recovery of EPSC amplitudes given as the ratio between the EPSC amplitude after and before a train of 100 APs at 40 Hz (*n* = 9 cells each). **(K)** Mean responses measured by application of a 100 μM glutamate solution (*n* = 10 and 9 cells). (Bars in plots depict mean and SEM).

FIGURE S8 Immunostaining of transfected hippocampal cultures from Mover knockout neurons. **(A–C)** Distribution of the indicated recombinant Mover variants in Mover knockout cultures immunostained for GFP and Bassoon. The panels show representative examples underlying the quantitative data displayed in Figure 8D. Left: low magnification overviews. Bassoon (magenta) and GFP (green) immunofluorescence are overlayed. Middle: zooms of the boxes. Arrows indicate examples of colocalization. Right: single-fluorescence images of the areas containing arrows.
