# Supplementary figures and images for: The Calmodulin Binding Region of the Synaptic Vesicle Protein Mover Is Required for Homomeric Interaction and Presynaptic Targeting
Source: Front Mol Neurosci. 2019 Nov 8;12:249. doi: 10.3389/fnmol.2019.00249 (PMC6856015; doi:10.3389/fnmol.2019.00249)

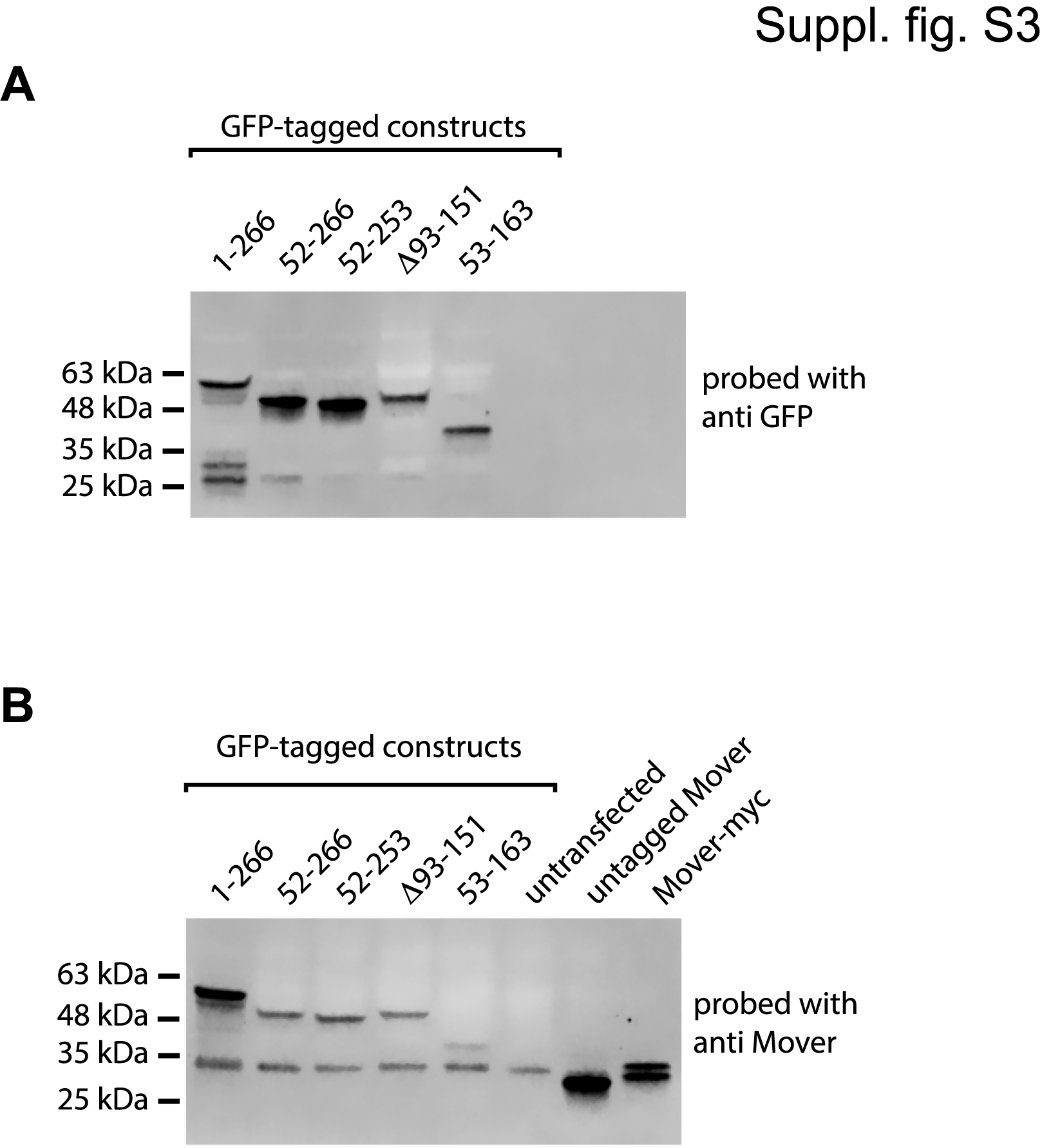

Supplement: FIGURE S1 — Triple immunostaining of synaptically targeted constructs for quantitative analysis. (A–C) Fluorescence microscopy of GFP-tagged rat Mover constructs and GFP tagged VAMP (a marker of synaptic vesicles) in DIV14 rat hippocampal neurons. The inverted gray level images represent GFP fluorescence detected with a 20× objective. The cultures were triple stained for GFP, the synaptic vesicle marker Synaptophysin and the dendrite marker MAP2. A 40× objective was used to record triple fluorescence images. Examples of such triple fluorescence images are shown in the small panels, and the boxes indicate where these examples are located in the 20× images. For quantitative analysis of the synaptic accumulation of the recombinant proteins the average fluorescent intensity of a synaptically localized punctate GFP-signal was divided by the average GFP-fluorescence intensity of a nearby axonal region. Punctate GFP-signals were defined as synaptic if they colocalized with the synapse marker Synaptophysin at a dendrite, identified by MAP2. (D) The ratio of synaptic vs. non-synaptic GFP-fluorescence, representing the enrichment of the protein at synapses compared to non-synaptic sites, of Mover-mGFP and 52-266-mGFP was similar to the enrichment of the presynaptic marker GFP-VAMP (n = 6 regions of interest, each representing 10 synaptic and 10 non-synaptic sites. N = 2 independent experiments. One-way ANOVA test; p = 0.32). [file Image_1.tif]

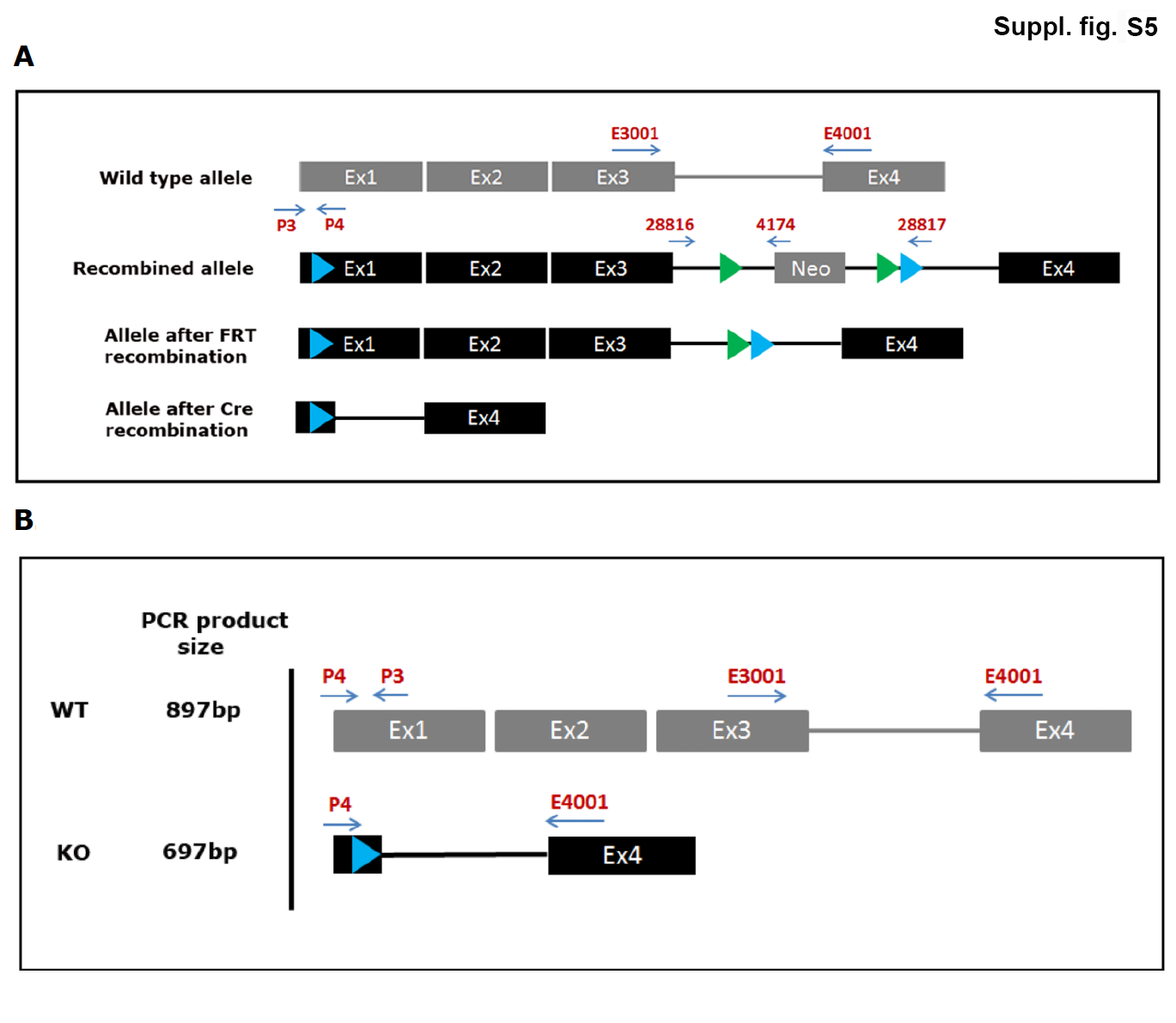

Supplement: FIGURE S2 — Triple immunostaining of homogeneously distributed constructs. (A–C) Fluorescence microscopy of GFP-tagged rat Mover constructs in DIV14 rat hippocampal neurons, indicating that these recombinant proteins are homogeneously distributed. The areas indicated by the boxes are zoomed in the small panels. Arrowheads indicate examples of axons, identified as GFP-positive and MAP2-negative processes. The constructs are homogeneously distributed in the axons, even when the axon contacts a dendrite. The punctate Synaptophysin staining indicates that synapses do exist in the cultures. The distribution of each construct was determined in more than 30 neurons on a total of six coverslips from three independent experiments (the same neurons and experiments as in Figure 1). [file Image_2.tif]

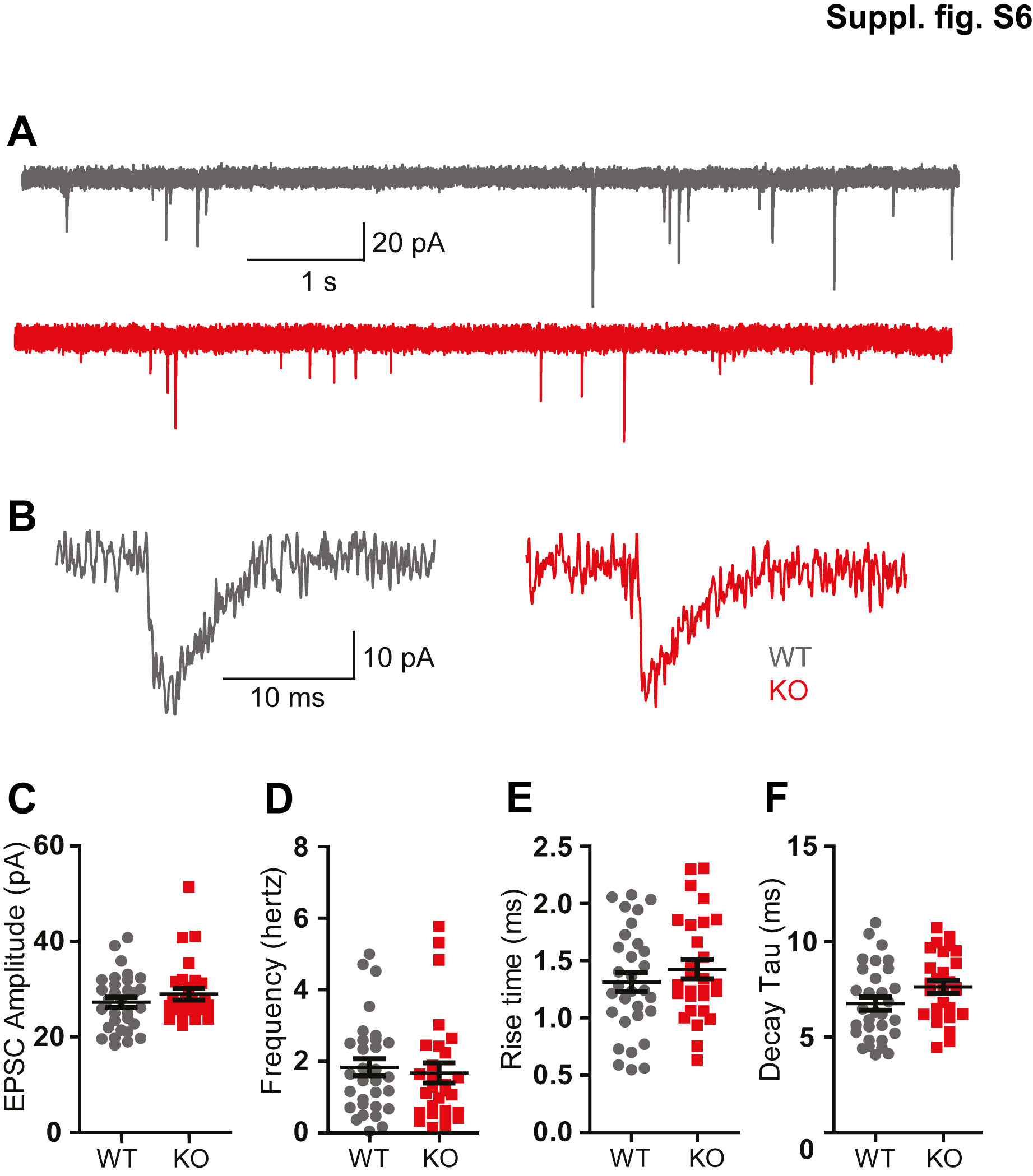

Supplement: FIGURE S3 — Characterization of recombinant Mover constructs expressed in HEK293 cells. Lysates from untransfected HEK293 cells and HEK293 cells expressing one of the Mover constructs were analyzed by western blotting. (A) Western Blot of lysates from cells expressing the indicated constructs, probed with rabbit anti-GFP. Based on their amino acid sequence the following molecular weights of the GFP-fusion proteins are expected: 57 kDa for 1–266, 52 kDa for 52–266, 50 kDa for 53–253, 52 kDa for Δ93–151, and 40 kDa for 53–163. The observed bands correspond to these molecular weights. In addition, 1–266 and, to a smaller extent, 52–266 produce smaller molecular weight bands, probably representing proteolytic degradation products. (B) Western Blot of lysates from cells expressing the indicated constructs, probed with rabbit anti-Mover. All constructs are detected by the Mover antibody and display the predicted molecular weights. In addition, the Mover antibody detects a ca. 32 kDa band in untranstected cells, which may correspond to the human Mover variant FAM79A. Mover-myc runs appears as double band. Both bands have higher molecular weight than untagged recombinant Mover. The data represent three independent experiments, i.e., three HEK293 cell transfections followed by lysis. [file Image_3.tif]

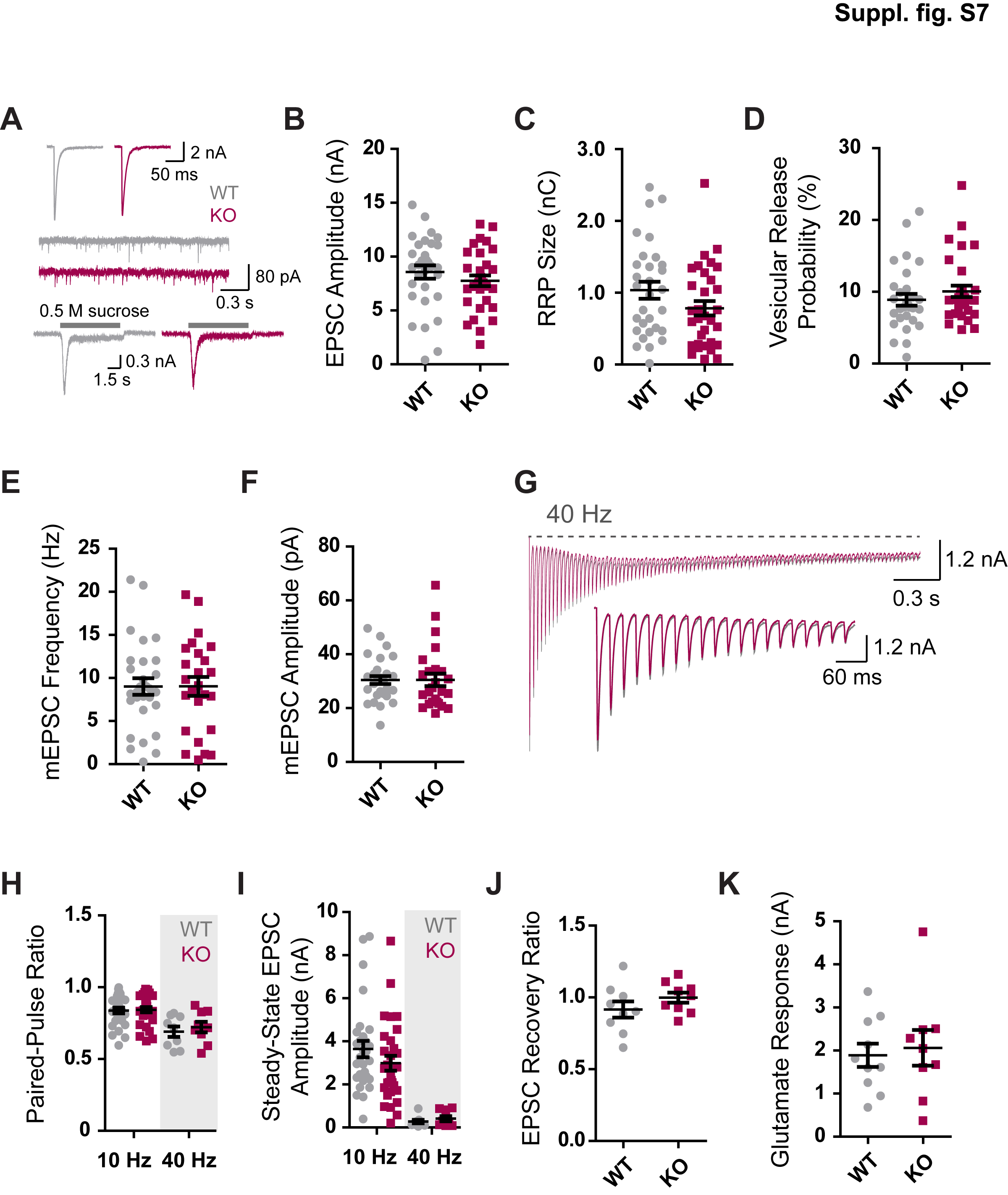

Supplement: FIGURE S4 — Helical wheel projections of CaM-binding peptides derived from Mover (A) and bMunc13-2 (B). For the generation of CaM-insensitive Mover, a similar strategy was applied as previously established in bMunc13-2. In addition to the replacement of the hydrophobic anchor residue F4 in Mover(203-221) by a charged Arg residue, the Lys residues forming the basic patch at the opposite site of the presumed alpha-helix (K5, K13, K17) were replaced by acidic Glu residues. As Lys and Glu have similar propensities to form an alpha helix, the charge of this helix patch was switched without changing the overall secondary structure. [file Image_4.tif]

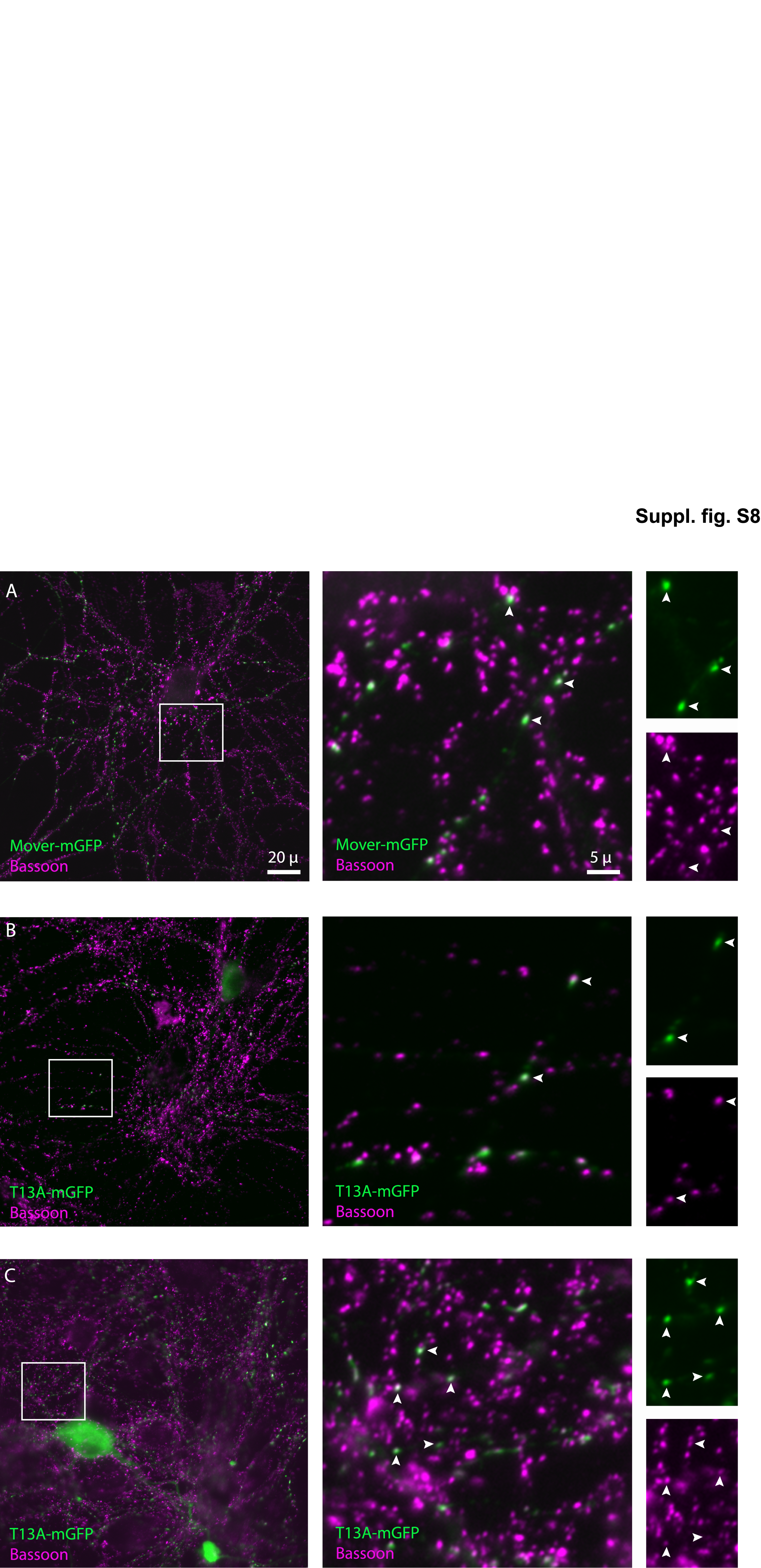

Supplement: FIGURE S5 — Knockout strategy. Schematic representation of the knockout strategy (A). The entire Mover gene including exons and introns spans 3616 base pairs. Blue triangles represent loxP sites. Green triangles represent FRT sites used to remove the Neo cassette via FLP recombinase. The 5’ loxP site is located upstream of the translation start site in the 5’ UTR region of exon 1. Cre mediated excision removes exons 1 through 3 and part of the downstream intron.Three primers (P4, E3001 and E4001) were used for genotyping (B). A PCR reaction including all three primers produces a 867 bp product on wildtype DNA (generated by P4 and P3), a 697 bp product on knockout DNA (generated by P4 and E4001), and both products on DNA from heterozygous animals. [file Image_5.tif]

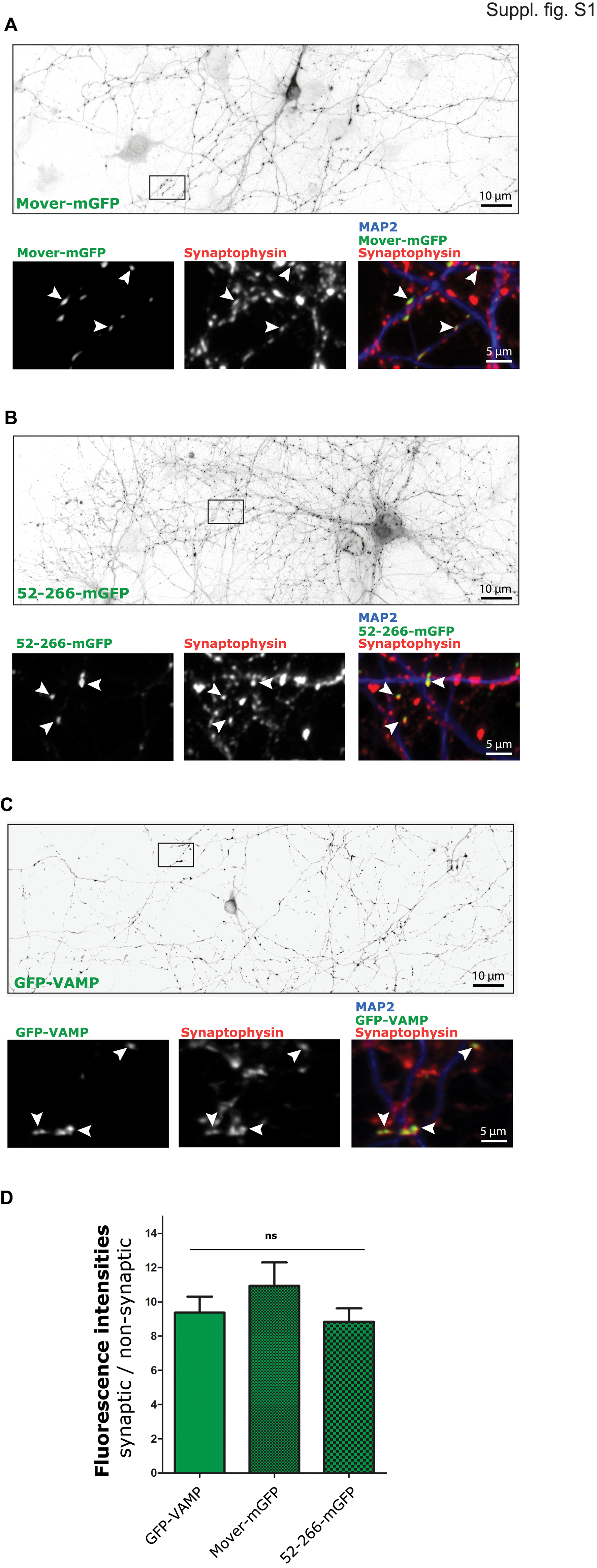

Supplement: FIGURE S6 — Spontaneous transmission in cultured neurons is unchanged in the absence of Mover. Whole cell recordings of miniature EPSCs in hippocampal dissociated cultures measured in the presence of 1 μM TTX. (A,B) Representative current traces of mEPSC recordings in WT (n = 31 cells) and Mover KO cultured neurons (n = 28 cells). (C) mEPSC amplitude, (D) frequency, (E) 10–90 rise time, and (F) time constant of decay are unchanged. Error bars represent SEM. [file Image_6.tif]

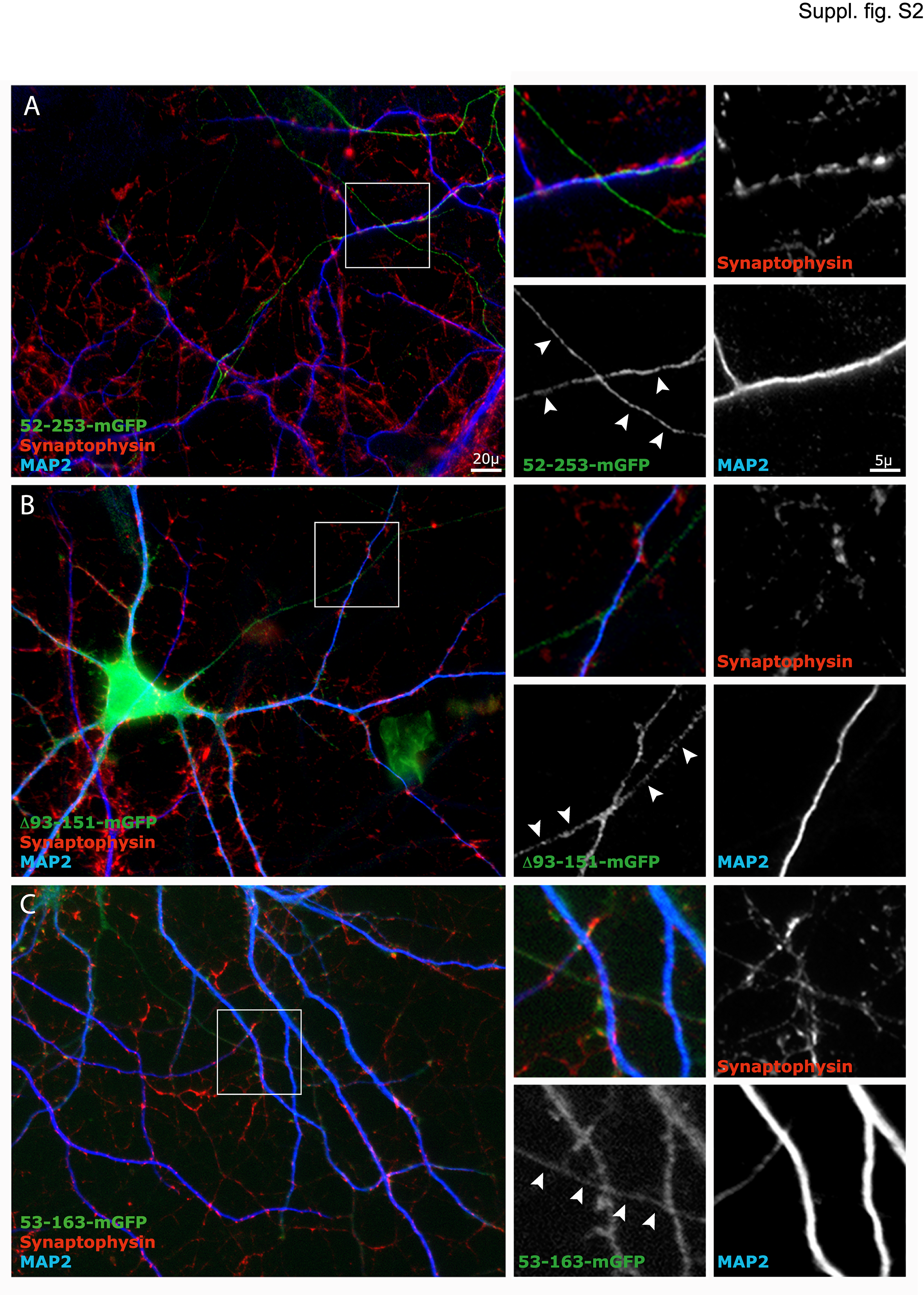

Supplement: FIGURE S7 — Synaptic transmission is not altered in cultured autaptic hippocampal neurons from Mover KO mice. (A) Representative traces of AP-evoked EPSCs (i) spontaneously occurring mEPSCs (ii) and sucrose-evoked EPSCs in cultured autaptic neurons. (B) Mean amplitude of AP-evoked EPSCs (n = 31 and 32 cells). (C) RRP size measured in response to 0.5 M sucrose (n = 30 and 32 cells) (D) Mean vesicular release probability calculated by dividing the charge of AP-evoked EPSCs by the charge of sucrose evoked responses (n = 30 and 32 cells). (E) and (F) Mean amplitude and frequency of mEPSCs measured in presence of 300 nM TTX for 100s. (n = 29 and 25 cells). (G) Averaged EPSC responses during a 40 Hz AP train (n = 9 cells each) (H) and (I) Paired-pulse ratio and steady-state EPSC responses during trains of APs at indicated frequencies (n = 29 and 30 cells for 10 Hz, n = 9 cells each for 40 Hz stimulation). (J) Recovery of EPSC amplitudes given as the ratio between the EPSC amplitude after and before a train of 100 APs at 40 Hz (n = 9 cells each). (K) Mean responses measured by application of a 100 μM glutamate solution (n = 10 and 9 cells). (Bars in plots depict mean and SEM). [file Image_7.tif]

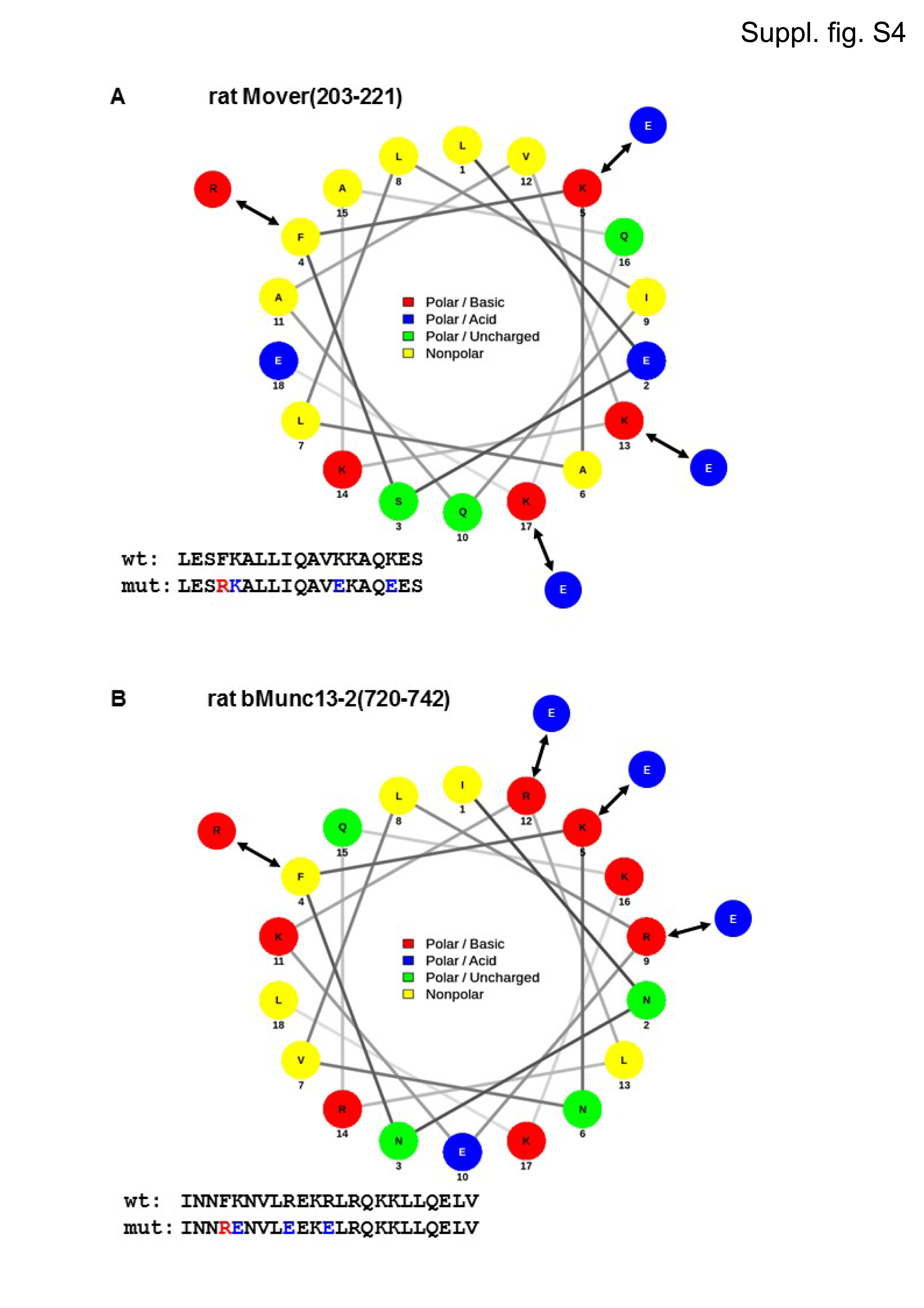

Supplement: FIGURE S8 — Immunostaining of transfected hippocampal cultures from Mover knockout neurons. (A–C) Distribution of the indicated recombinant Mover variants in Mover knockout cultures immunostained for GFP and Bassoon. The panels show representative examples underlying the quantitative data displayed in Figure 8D. Left: low magnification overviews. Bassoon (magenta) and GFP (green) immunofluorescence are overlayed. Middle: zooms of the boxes. Arrows indicate examples of colocalization. Right: single-fluorescence images of the areas containing arrows. [file Image_8.tif]
